# Advances in Pathogenesis, Progression, Potential Targets and Targeted Therapeutic Strategies in SARS-CoV-2-Induced COVID-19

**DOI:** 10.3389/fimmu.2022.834942

**Published:** 2022-04-05

**Authors:** Hong Zhou, Wei-Jian Ni, Wei Huang, Zhen Wang, Ming Cai, Yan-Cai Sun

**Affiliations:** ^1^ Department of Pharmacy, Anhui Provincial Cancer Hospital, The First Affiliated Hospital of USTC, Division of Life Sciences and Medicine, University of Science and Technology of China, Hefei, China; ^2^ Inflammation and Immune Mediated Diseases Laboratory of Anhui Province, The Key Laboratory of Anti-inflammatory of Immune Medicines, Ministry of Education, Anhui Institute of Innovative Drugs, School of Pharmacy, Anhui Medical University, Hefei, China; ^3^ Anhui Provincial Hospital, The First Affiliated Hospital of USTC, Division of Life Sciences and Medicine, University of Science and Technology of China, Hefei, China; ^4^ The Third People’s Hospital of Hefei, The Third Clinical College of Anhui Medical University, Hefei, China; ^5^ Anhui Provincial Children’s Hospital, Children’s Hospital of Fudan University-Anhui Campus, Hefei, China; ^6^ Department of Pharmacy, The Second Affiliated Hospital of Anhui University of Chinese Medicine, Hefei, China; ^7^ School of Pharmacy, Anhui University of Chinese Medicine, Hefei, China

**Keywords:** severe acute respiratory syndrome coronavirus 2 (SARS-CoV-2), coronavirus disease 2019 (COVID-19), small molecular inhibitor, vaccine, traditional Chinese medicine, potential target, targeted therapeutic strategy

## Abstract

As the new year of 2020 approaches, an acute respiratory disease quietly caused by severe acute respiratory syndrome coronavirus 2 (SARS-CoV-2), also known as coronavirus disease 2019 (COVID-19) was reported in Wuhan, China. Subsequently, COVID-19 broke out on a global scale and formed a global public health emergency. To date, the destruction that has lasted for more than two years has not stopped and has caused the virus to continuously evolve new mutant strains. SARS-CoV-2 infection has been shown to cause multiple complications and lead to severe disability and death, which has dealt a heavy blow to global development, not only in the medical field but also in social security, economic development, global cooperation and communication. To date, studies on the epidemiology, pathogenic mechanism and pathological characteristics of SARS-CoV-2-induced COVID-19, as well as target confirmation, drug screening, and clinical intervention have achieved remarkable effects. With the continuous efforts of the WHO, governments of various countries, and scientific research and medical personnel, the public’s awareness of COVID-19 is gradually deepening, a variety of prevention methods and detection methods have been implemented, and multiple vaccines and drugs have been developed and urgently marketed. However, these do not appear to have completely stopped the pandemic and ravages of this virus. Meanwhile, research on SARS-CoV-2-induced COVID-19 has also seen some twists and controversies, such as potential drugs and the role of vaccines. In view of the fact that research on SARS-CoV-2 and COVID-19 has been extensive and in depth, this review will systematically update the current understanding of the epidemiology, transmission mechanism, pathological features, potential targets, promising drugs and ongoing clinical trials, which will provide important references and new directions for SARS-CoV-2 and COVID-19 research.

## Introduction

To date, the 2019 coronavirus disease (COVID-19) pandemic caused by severe acute respiratory syndrome coronavirus 2 (SARS-CoV-2) has infected 440 million people and caused approximately 5.97 million deaths, and these data are still growing rapidly (https://coronavirus.jhu.edu/map.html). This terrible disease not only causes a large number of casualties, but also seriously affects the world economy and peaceful development (1). Therefore, elucidating the possible mechanisms and potential targets of the disease and exploring effective therapeutic drugs and strategies are the most urgent efforts worldwide.

Studies have confirmed that SARS-CoV-2 is a single-stranded RNA-positive Sarbecovirus subgenus β-coronavirus (2). Homology analysis found that the genome sequence of SARS-CoV-2 is approximately 79% homologous with that of the previous SARS-CoV, and more than 50% homologous with that of MERS-CoV, which provides a certain basis and direction for its research (3). However, due to the extremely unstable genetic material of SARS-CoV-2, it is prone to mutations, producing mutant strains or promoting rapid virus evolution (**Table 1**), promoting the continued progress of COVID-19 and a wave of turbulence. This once again threatens the prevention and research of COVID-19 (39). Therefore, the need for targeted drugs and promising treatment strategies is urgent.

**Table 1:** Characteristics of the current concerned SARS-CoV-2 variant strains.

Variant Name	Discovery Time	Original Location	Epidemiological Characteristics	Reference
B.1.1.7 (Alpha)	September 2020	United Kingdom	The infectivity of this type of variant strain has changed, and the transmission speed has increased by approximately 50%; the sensitivity to monoclonal antibody therapy remains unchanged; it can be effectively neutralized by vaccines or antibodies produced by natural infections	Sabino et al. (4) Thye et al. (5)
B.1.351 (Beta) B.1.351.2 B.1.351.3	May 2020	South Africa	The infectivity of this variant strain increases by approximately 50%; the sensitivity to monoclonal antibody treatment is reduced; the neutralizing effect of antibodies produced by vaccines or natural infections is also significantly reduced	Martin et al. (6) Benton et al. (7)
B.1.1.28.1 (P.1/Gamma)	November 2020	Japan/Brazil	This type of variant strain is less sensitive to monoclonal antibody therapy; the neutralizing effect of antibodies produced by vaccines or natural infections is also significantly reduced	Faria et al. (8) Hemmer et al. (9)
B.1.617.1 (Kappa) B.1.617.2 (Delta)	October 2020	India	The infectivity of this type of variant strain is enhanced; the sensitivity to monoclonal antibody therapy may be reduced; the neutralizing effect of antibodies produced by vaccines or natural infections may be reduced	Mishra et al. (10) Kannan et al. (11)
B.1.1.28.2 (P.2/Zeta)	April 2020	Brazil	Potential depletion in neutralization by convalescent and postvaccination sera or monoclonal antibody treatments	Sapkal et al. (12) Zhang et al. (13)
B.1.427/B.1.429 (Epsilon)	June 2020	United States	The mutant strain has enhanced toxicity and immune escape ability, resulting in low efficacy or even ineffectiveness of various serum vaccines and neutralizing antibodies, ~20% increased transmissibility	McCallum et al. (14) Deng et al. (15)
C.37 (Lambda)	December 2020	Peru	This mutant strain will affect the effectiveness of vaccines and neutralizing antibodies, and is believed to promote the virus to invade host cells and help the virus escape the host immune system	Romero et al. (16) Darvishi et al. (17)
B.1.621 (Mu) B.1.621.1	January 2021	Colombia	The mutant strain is highly resistant to COVID-19 convalescent serum and vaccines vaccinated thus far, with enhanced transmission and pathogenicity, and is likely to have immune escape and natural derivation capabilities	Laiton-Donato et al. (18) Uriu et al. (19)
B.1.1.28.3 (P.3/Theta)	March 2021	Philippine	The mutant strain may show stronger transmission, while reducing the neutralization of vaccine and convalescent serum	Shuai et al. (20) Moubarak et al. (21)
B.1.1.523	May 2020	Russia	The enhanced immune escape ability of the mutant strain leads to weakened vaccine effectiveness	van der Veer et al. (22)
C.1.2	March 2021	South Africa	The mutation degree of this mutant strain far exceeds that of other strains, the gene mutation rate is higher but the incidence rate is low, and the infectivity and immune escape ability are enhanced	Albayat et al. (23) Yang et al. (24)
R.1	January 2021	Japan	This variant strain is easier to spread and may have the ability to actively evade vaccine antibodies	Nagano et al. (25) Sekizuka et al. (26)
C.36.3	January 2021	Thailand-Egypt	This strain has been listed by the WHO as a “mutant strain under surveillance”, which means that the strain is potentially dangerous	https://www.who.int/en/activities/tracking-SARS-CoV-2-variants/
B.1.1.519	November 2020	Mexico	This variant strain reduces the activity of some monoclonal antibodies, but does not show changes in immune escape ability and pathogenicity	Rodríguez-Maldonado et al. (27)
B.1.1.318	February 2021	United Kingdom	This variant strain is highly transmissible and may impair the efficacy of the vaccine	Laine et al. (28) Manouana et al. (29)
B.1.466.2	November 2020	Indonesia	This mutant strain has a high infection rate in Indonesia (approximately 48%), but the overseas infection rate is low (<0.5%)	Fibriani et al. (30) Sam et al. (31)
B.1.620	February 2021	Europe	This mutant strain carries mutations and missing information of a variety of strains of interest, and is likely to have antibody-mediated immune escape. It may be ineffective against mRNA vaccines and is widely spread in central Africa.	Dudas et al. (32) Zahradník et al. (33)
B.1.526 (Iota)	November 2020	United States	The mutant strain has a faster transmission speed and a higher lethality rate, is partially or completely resistant to monoclonal antibodies, and is not sensitive to the neutralization effect of plasma and serum during the recovery period.	Annavajhala et al. (34) Thompson et al. (35)
B.1.525 (Eta)	December 2020	Nigeria/United Kingdom	The mutant strain has strong transmission and immune escape ability, which can weaken the neutralization efficiency of vaccines and antibodies	Bugembe et al. (36)
B.1.630	March 2021	Dominican Republic	This mutant strain has a large number of spike protein mutation points, but weaker transmissibility than the Delta variant. It still needs attention	https://www.who.int/en/activities/tracking-SARS-CoV-2-variants/
B.1.1.529 (Omicron)	November 2021	South Africa Botswana	The mutant strain has more mutation sites and significantly enhanced infectivity, which is 10× and 2× higher than the original virus or Delta mutant strain, respectively; the immune escape ability is enhanced and twice that of the Delta mutant strain, resulting in a decreased efficiency of monoclonal antibodies and resistant to vaccines; the speed of virus infection has increased, and there is an increased risk of reinfection	Abdool and de Oliveira (37), Chen et al. (38)

In view of this, this article will comprehensively analyze the epidemiological and pathological characteristics of SARS-CoV-2 to promote further research on COVID-19. In-depth discussion of promising therapeutic targets and possible pathogenesis during SARS-CoV-2 infection will accelerate the development of promising drugs, including small molecule drugs, vaccines and biological products, traditional Chinese medicines (TCMs) and symptomatic drugs, and the exploration of effective treatment strategies will eventually promote their clinical applications to overcome SARS-CoV-2-induced COVID-19.

## Structural Information, Epidemiology and Pathology Features of SARS-CoV-2

**Figure 1:**
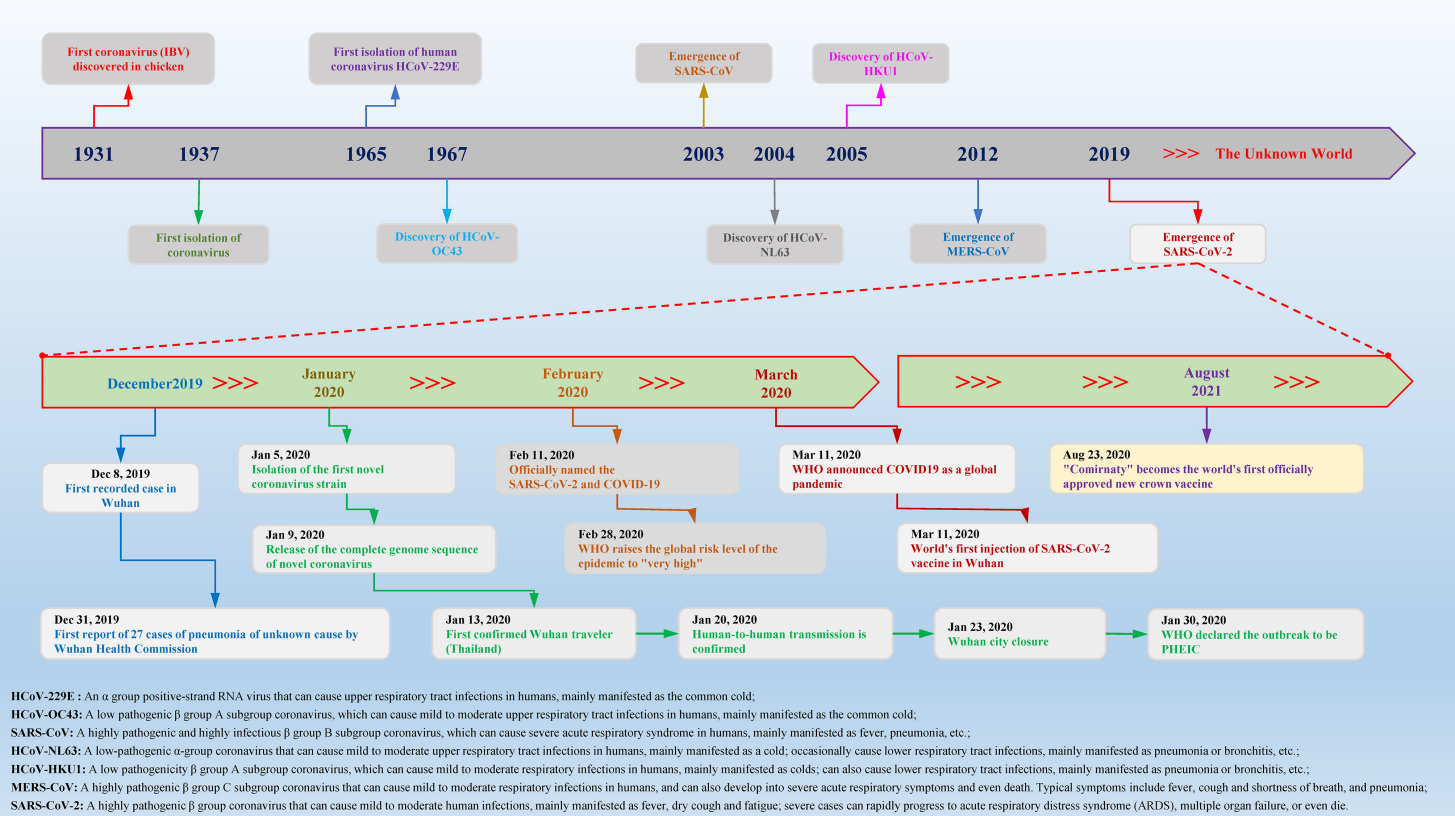
Timeline of key events for coronavirus discovery and research. Coronavirus was first isolated from chickens in 1937. With the passage of time and changes in the environment, in the past 84 years, a variety of different species and subgroups of coronaviruses have been discovered, identified, named, and researched. In December 2019, Wuhan, China, reported a novel coronavirus case for the first time. In a short period of time, the COVID-19 epidemic caused by SARS-CoV-2 spread to the world and caused major disasters and epidemics. In the past two years, there have been more than 440 million confirmed cases worldwide, causing approximately 5.97 million deaths, which has caused great social upheavals and dangers.

**Figure 2:**
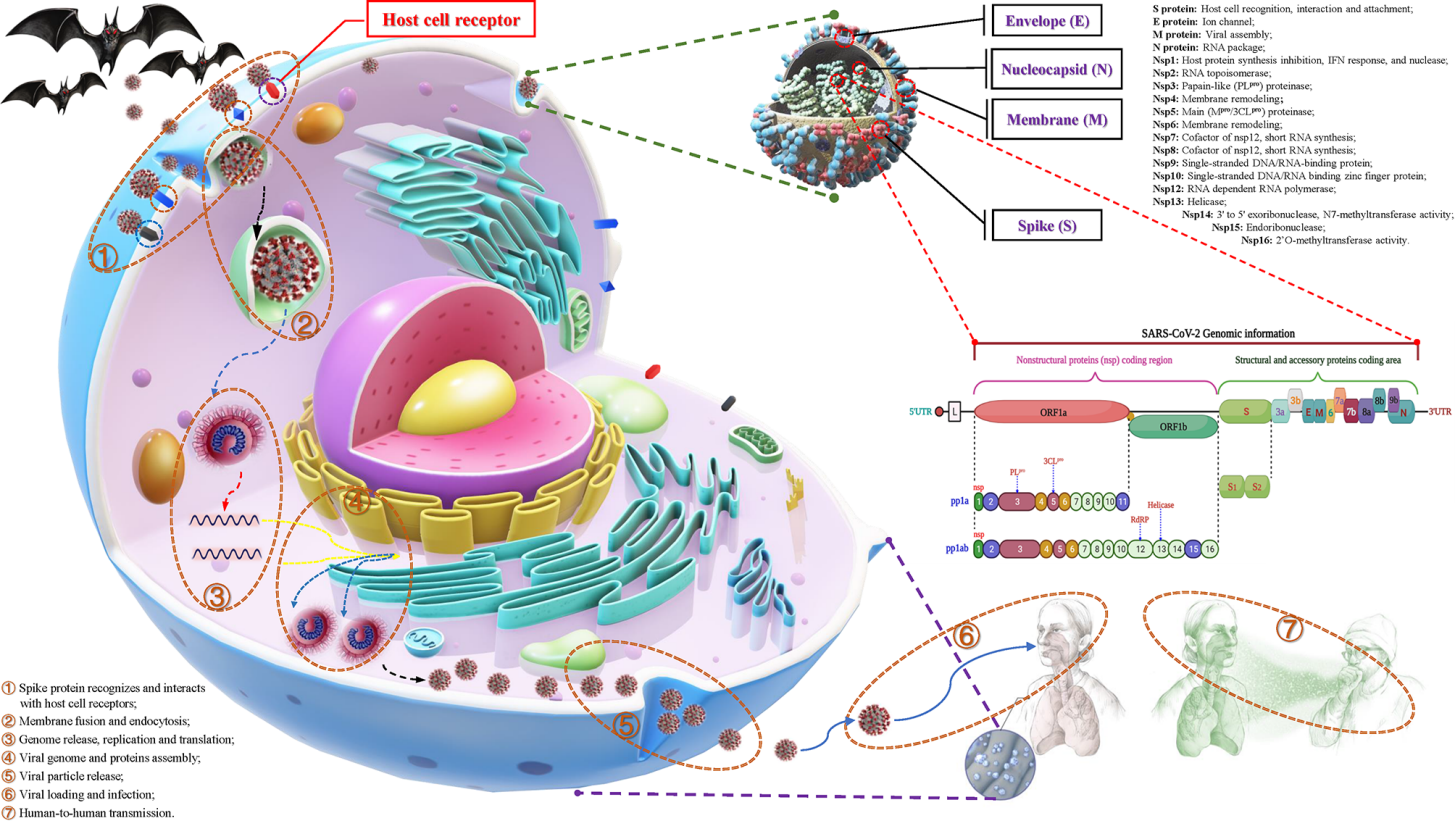
The structural features, potential functions and transmission process of SARS-CoV-2. Structurally, the outer side of SARS-CoV-2 is surrounded by a capsid, which is mainly composed of spike (S), membrane (M), and envelope (E) proteins, while the nucleocapsid (N) protein is accompanied by the genome. The genomic structure of SARS-CoV-2 is based on a single-stranded positive-stranded RNA, which contains a 5’-methylated cap and a 3’-polyadenylic acid tail, arranged in the following order: 5’-end; nonstructural protein (nsp) coding region [open reading frame (ORF1a/b)]; structure and accessory protein coding regions such as S, E, M, N and 3a, 3b, 6, 7a, 7b, 8a, 8b, 9b. Among them, the open reading frame (ORF) 1a/b is responsible for encoding a variety of nonstructural proteins, mainly RNA-dependent RNA polymerase (RdRP), papain-like protease (PL^pro^) and 3C-like protease (3CL^pro^). The putative functions of these proteins are mentioned in the figure. During the infection process, SARS-CoV-2 recognizes and interacts with host cell surface receptors and enters the host cell through membrane fusion and endocytosis. After entering the host cell, SARS-CoV-2 releases its genome and translates a large number of nsps, including RdRP, PL^pro^ and 3CL^pro^. Under the action of these enzymes, it synthesizes the new RNA genome and assembles to form virus particles, which are then released into the extracellular space through exocytosis. Uncontrolled replication promotes SARS-CoV-2 infection, leading to immune disorders and inflammatory cytokine storms and ultimately leading to damage to multiple organs, especially the lungs.

According to statistics, there are currently two types (highly pathogenic and minimally pathogenic) of six coronaviruses (CoVs) that can cause human diseases. Among them, highly pathogenic CoVs, including SARS-CoV (Guangdong, China, 2002), Middle East respiratory syndrome coronavirus (MERS-CoV, Saudi Arabia, 2012) and the existing SARS-CoV-2 can cause severe human lung infections and multiple organ dysfunctions (40). The specific development trend of these CoVs is included in **Figure 1**. With the help of the latest omics, structural biology and other technologies, researchers have initially mastered the genome and structural information of SARS-CoV-2 (41). Specifically, the structure of SARS-CoV-2 is composed of the nucleocapsid (N) protein wrapped with RNA as genetic material located in the core region, accompanied by spike (S) protein, envelope (E) protein and membrane (M) protein scattered in the peripheral area, and the genome structure is mainly composed of multiple open reading frames (ORFs) (42). According to the current gene bank annotation (NC_045512.2), 2 functional ORFs (ORF1a and ORF1b) are translated into replicase complexes, and 4 functional ORFs encode S, E, M and N proteins in the 5’-3’ direction, while the remaining ORFs are distributed in the abovementioned functional genes, encoding multiple accessory proteins, including 3a/3b, 6, 7a/7b, 8a/8b and 9b (43). Further research found that the ORF1a- and ORF1b-translated viral replicase/transcriptase protein complex is cleaved to form up to 16 kinds of nonstructural proteins (nsps) by the virus/host proteolytic enzymes, including 3C-like main protease (3CL^pro^ or M^pro^) and papain-like protease (PL^pro^) (44). During this process, PL^pro^ cuts the N-terminus of the polyprotein to form nsp1, nsp2 and nsp3, which are required for SARS-CoV-2 replication, while 3CL^pro^ cleaves and separates the polyprotein pp1ab to generate nsp4-16 to form multiple active proteins, including RdRp and helicases, which are essential requirements for the life cycle of SARS-CoV-2 in host cells (45). The useful information shown in **Figure 2** will help scientists better discover potential targets that interfere with the replication, spread, and pathogenicity of SARS-CoV-2 and develop promising vaccines, small molecule drugs and TCMs that can be used in the clinic.

Meanwhile, the initial epidemiological research results indicate that SARS-CoV-2 spreads from person to person mainly through the respiratory tract, droplets or aerosols (46). However, based on multiple studies, it can be seen that SARS-CoV-2 can be spread not only through the abovementioned channels but also through other means, which is mainly manifested as follows: 1. There have been cases showing that SARS-CoV-2 can spread by the placenta, but vertical transmission rarely occurs. 2. According to existing research, the virus can spread among minks and can infect humans. Meanwhile, cats and ferrets have been confirmed to be able to transmit to each other, but there are no reported cases of transmission to humans. 3. There have been studies speculating that this virus can also be spread by direct contact and pollutants, but this may be just an unusual route of transmission. 4. Although live virus has been isolated from saliva and feces, viral RNA has also been detected in semen and blood transfusions (47). There are currently no reports of sexual or blood transmission and only one report of possible fecal-respiratory transmission (48), which will provide us with important guidance for all-round protection.

**Figure 3:**
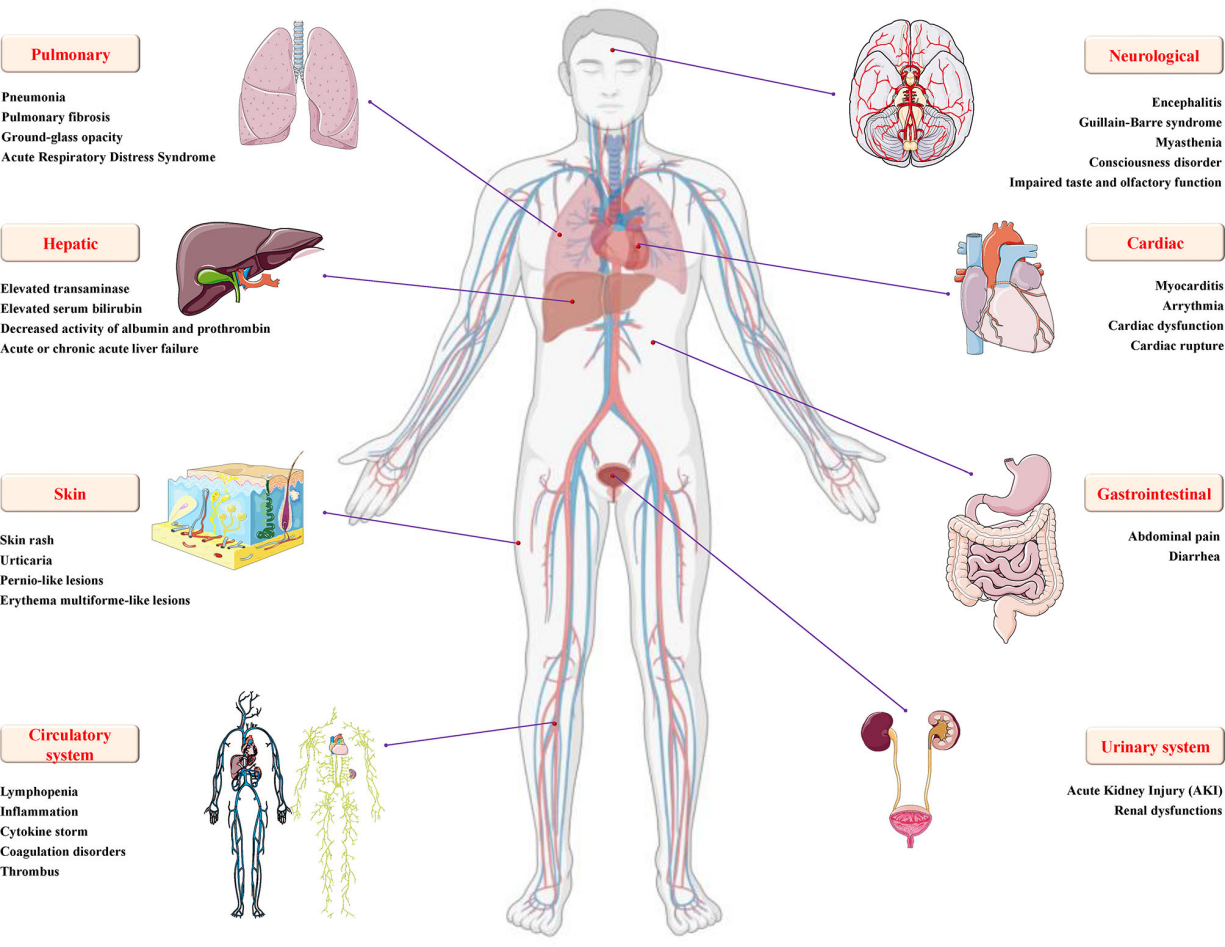
Details of multiple organ injury caused by SARS-CoV-2. In addition to varying degrees of pulmonary inflammation, embolism, and acute respiratory distress syndrome, COVID-19 caused by SARS-CoV-2 infection can also cause various organ dysfunctions and damages, including but not limited to encephalitis, Gillan-Barre syndrome, muscle weakness and other nervous system dysfunction; increased blood pressure, arrhythmia, myocardial ischemia, cardiac insufficiency, rupture and other cardio/cerebrovascular system damage; urogenital system damage, such as glomerular congestion and acute kidney injury; digestive system damage, such as diarrhea, increased transaminase/serum bilirubin, decreased albumin/prothrombin activity, acute or chronic acute liver failure, and skin and circulatory diseases, such as skin rash, urticaria, pernio-like lesions, inflammation, cytokine storm, coagulopathy and thrombosis.

Researchers conducted a systematic analysis of SARS-CoV-2-infected patients and found that almost all patients had frosted glass shadows on both sides of their lungs (49). The initial symptoms of the patient mainly included fever, cough and sputum, hemoptysis, headache and myalgia or fatigue, diarrhea, dyspnea, etc. As the disease progresses, symptoms such as inflammation, fibrosis and edema appear in the lungs, which gradually develop into acute respiratory distress syndrome (ARDS) and cause lung failure (50). Meanwhile, SARS-CoV-2 infection also causes damage to multiple organ functions, including digestive system injury, such as liver degeneration and spot necrosis, and the epithelium of the esophagus, stomach and intestine mucosa show varying degrees of degeneration, necrosis and exfoliation; brain and nervous system damage, such as cerebral congestion and edema, some neuronal degeneration and ischemic changes; cardiovascular system damage, such as increased blood pressure and arrhythmia, increases the probability of myocardial infarction, causes myocardial ischemia, necrosis, thrombosis and cardiac insufficiency; genitourinary system damage, including glomerular congestion, segmental hyperplasia or necrosis, protein exudation in the glomerular capsule, and acute kidney injury (**Figure 3**); and some patients still die after treatment (51). Based on this, being familiar with the pathological changes caused by SARS-CoV-2 will lay the foundation for clinical diagnosis and targeted therapy.

At present, new cases of COVID-19 are caused by multiple SARS-CoV-2 variants in many countries (52). Currently, a number of major variants are rapidly growing and causing concern, including alpha (B.1.1.7), beta (B.1.351), gamma (B.1.1.28.1), delta (B.1.617.2) and omicron (B.1.1.529), and the characteristics of these variants are shown in **Table 1**. Meanwhile, different mutant strains have different characteristics. For example, the gamma variants increase toxicity and increase the risk of hospitalization and death, while Delta strains are highly infectious and spread quickly, especially the shortened incubation period or passage interval, which increases the risk of global epidemics (10, 53). Authoritative research shows that SARS-CoV-2 has evolved more than 800 different subtypes or branches, and its variants may have exceeded 1,000 (54). In general, the direction of the mutation and evolution of the new coronavirus is mainly to break through immunity, avoid vaccines, increase exponential replication, and be highly infectious (37). Although the mutant strains are terrible, their diversity, transmission, epidemic, and pathogenic characteristics will provide important clues for the in-depth study of virus mutation mechanisms, exploration of novel potential targets, and development of effective vaccines, drugs, and therapeutic strategies.

## Potential Therapeutic Targets of SARS-CoV-2

**Figure 4:**
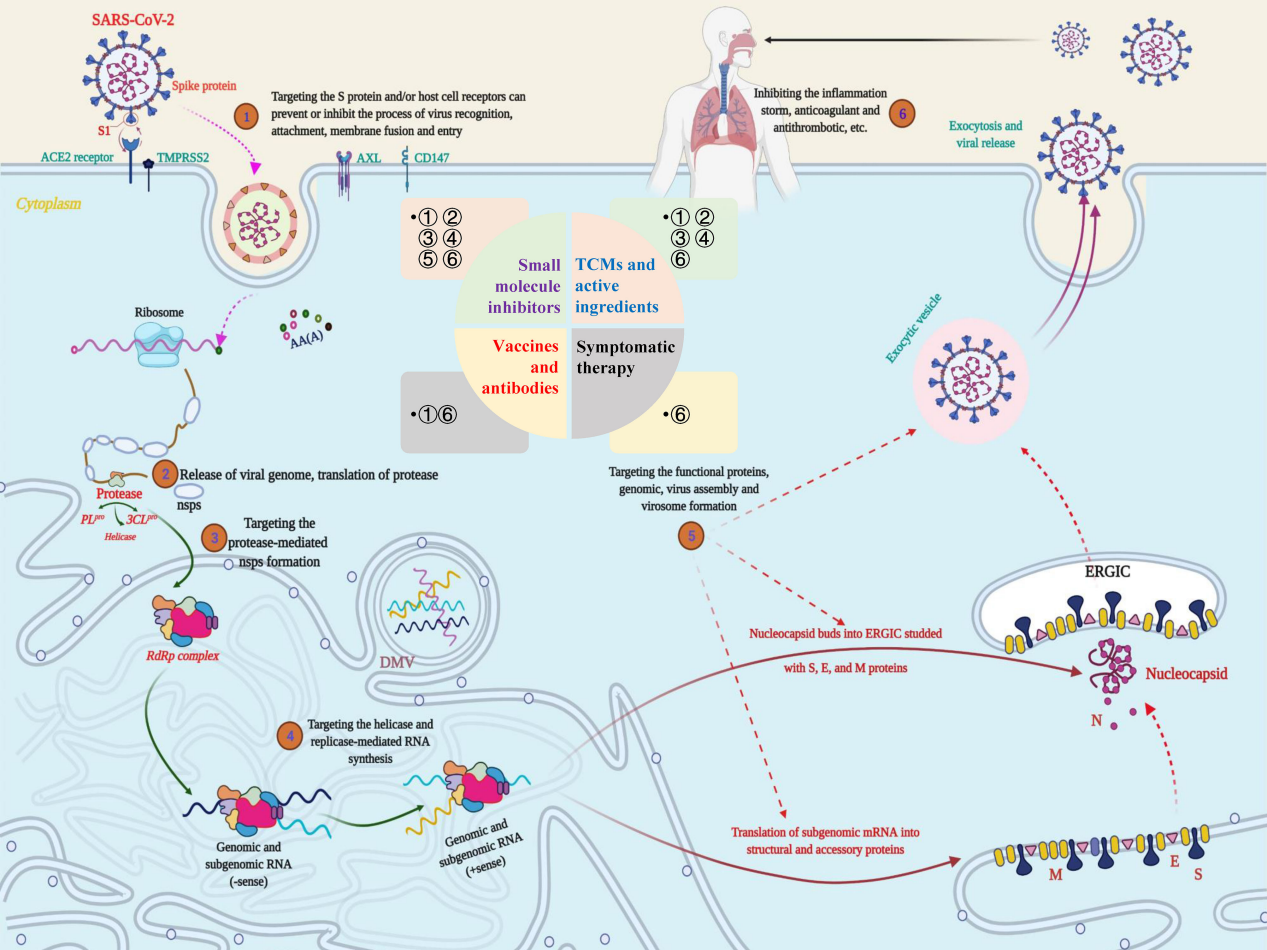
Potential targets and targeted therapeutic strategies for combating SARS-CoV-2-induced COVID-19. Scheme of the potential targets, intervention strategies and types of therapeutic drugs in the cycle of SARS-CoV-2 infection, replication, and transmission. During the infection stage, SARS-CoV-2 recognizes and interacts with host cell surface receptors through the spike (S) protein or transmembrane glycoprotein CD147 and enters the host cell through membrane fusion and endocytosis. After the virus enters the host cell, SARS-CoV-2 releases its nucleocapsid and genome into the cytoplasm and translates a large number of nonstructural proteins (nsps) including coding RNA-dependent RNA polymerase (RdRP), papain-like protease (PL^pro^) and 3C-like protease (3CL^pro^). Under the action of these enzymes, a full-length negative antisense genome template is synthesized to produce the new RNA genome and assembled to form virus particles, which are then released into the extracellular space through exocytosis. Uncontrolled replication promotes SARS-CoV-2 infection, leading to immune disorders and inflammatory cytokine storms and ultimately leading to damage to multiple organs, especially the lungs. The whole process exposed multiple potential targets, providing important guidance for research on anti-SARS-CoV-2 targets, drugs and treatment strategies.

Combining the research experience of SARS- and MERS-CoV to explore the potential therapeutic targets of SARS-CoV-2, the following aspects should be considered: enzymes and functional proteins that affect RNA synthesis and viral replication; structural proteins that affect virus entry and the self-assembly process; virulence factors that affect the host immune regulation; and host cell surface proteins and receptors (**Figure 4**). Correspondingly, therapeutic strategies are also divided into targeting SARS-CoV-2 and targeting host cells and the body’s immune system (55). Authoritative research shows that SARS‐CoV‐2 can encode a variety of proteins, including nsps, structural proteins, and several virulence factors (56). Moreover, multiple specific host cell surface receptors, coreceptors, and auxiliary proteases, including angiotensin converting enzyme 2 (ACE2), transmembrane protease serine 2 (TMPRSS2), cluster of differentiation 147 (CD147) tyrosine-protein kinase receptor UFO (AXL) and nonmuscle myosin heavy chain IIA (MYH9) (38, 57, 58), have been identified. Obviously, these targets will be the most promising targets for fighting the COVID-19 outbreak caused by SARS-CoV-2.

### RNA Synthesis and Replication Protease Targets

Nsps have proven to be widely involved in SARS-CoV-2 recognition, entry, inheritance, replication, and infection. Together with their key biological functions and relatively clear structure and active site, the main nsps, including PL^pro^, 3CL^pro^, RNA-dependent RNA polymerase (RdRP) and helicase, have become the first batch of targets to be considered for the development of small molecular inhibitors (**Figure 4**) **(** 59).

3CL^pro^, the aforementioned nsp5, was found to cut 11 sites on the polyprotein body encoded by ORF1ab and then release mature nsp4-nsp16, which is crucial to the life cycle of SARS-CoV-2 (60). Structural analysis showed that the protease monomer mainly contains a domain I (residues 8-101) and a long loop connected domain II (residues 102-184) and a domain III (residues 185-200), and the active site is located in the gap between domains I and II (61). Mature research on the function, structure and active site of 3CL^pro^ makes it a powerful target for anti-SARS-CoV-2 drugs such as small molecules and peptide inhibitors.

Unlike 3CL^pro^, PL^pro^ mainly cuts from the N-terminus of the polyprotein to release nsp1, 2 and 3, which will affect the accuracy of SARS-CoV-2 replication. Research on MERS-/SARS-CoV suggests that it has a powerful role in antihost innate immunity (62). Moreover, homology analysis found that SARS-CoV-2 and SARS-CoV PL^pro^ share approximately 83% of the sequence at the protein level. Combined with its indispensable role in virus replication and infection, PL^pro^ should be a valuable target for SARS-CoV-2 inhibitor research. Meanwhile, the use of X-ray crystallography and other techniques to analyze the structure of PL^pro^ will further facilitate the study of PL^pro^ inhibitors against SARS-CoV-2 (63, 64).

In the RNA replication of CoVs, RdRP promotes their evolution by affecting the fidelity of replication and mutation rates to help them adapt to the environment or host cells (65). Homology analysis found that SARS-CoV and SARS-CoV-2 share approximately 82% of the homologous sequence at the genome level, while RdRP shares a sequence of more than an astonishing 96% at the protein level (66). These findings remind us that RdRP will become one of the most promising targets for the study and treatment of SARS-CoV-2. High-resolution structural analysis revealed that the functional domain of SARS-CoV-2 RdRP is located at the C-terminus of the protein, where there is a conserved Ser-Asp-Asp motif. At the RNA level, nsp8 can control the *de novo* synthesis of up to 6 nucleotides, which will provide primers for nsp12/RdRP RNA synthesis. Meanwhile, the nsp7-nsp8 complex can increase the activity of RdRP, which in turn affects its binding to RNA (67). All these studies provide valuable references and directions for research on anti-SARS-CoV-2 targeting RdRP.

SARS-CoV-2 helicase (nsp13) is a multifunctional nucleoside triphosphate (NTP)-dependent protein. Structural analysis revealed that helicase contains a metal binding domain (MBD) composed of 26 cysteine residues at the N-terminus and a helicase domain (Hel) consisting of a conserved motif at the C-terminus (68). Functional studies found that, helicase can unwind double-stranded (ds) DNA and RNA in an NTP-dependent manner along the 5’-3’ direction during (69). It was found that the sequence of the helicase of SARS-CoV-2 is conserved and indispensable and is an essential component of virus replication. Based on these studies, helicase is expected to become a viable target against SARS-CoV-2 infection.

### Structural Protein Targets

Based on the current research results, the spike protein is one of the most critical structural proteins of SARS-CoV-2, which forms a special flower crown structure on the outer surface of the virus in the form of a trimer. Meanwhile, studies have found that the spike protein can directly affect the recognition, receptor binding, interaction, and virus entry between SARS-CoV-2 and host cells to determine the tissue or host preference in the initial stage of infection (70). In the spike (S)-mediated infection process, certain proteases in the host cell, such as TMPRSS2, can cleave the spike protein into two subtypes, the S1 subunit and the S2 subunit. The responsibility of the S1 subunit is to recognize and bind to host cell surface receptors, while the main task of the S2 subunit is to mediate the virus-cell and cell membrane fusion process (71). From the perspective of the mechanism, the structural integrity, cleavage and activation of the S protein perform crucial roles during host cell invasion and virulence. Therefore, it will have far-reaching significance to develop drugs and vaccines that affect the viral spike protein or specific receptors on the host cell surface to prevent SARS-CoV-2 from entering and infecting. Except for the outermost spike protein, the N protein is a highly immunogenic phosphoprotein and also a core and highly conserved component of SARS-CoV-2 (72). In the process of virion assembly, N protein combines with viral genomic RNA to produce a spiral nucleocapsid and is related to viral genome replication and regulation of cell signaling pathways. During this process, the N-terminal domain (NTD) and C-terminal domain (CTD) are necessary structures for effective binding to viral RNA (73). Meanwhile, studies have pointed out that the E protein mainly affects the structural integrity and virulence of SARS-CoV-2 (74). In addition, these proteins also exhibit the interferon (IFN) antagonistic properties. In particular, the M protein can prevent the formation of the MAVS-traf3-tbk1 complex and antagonize the production of IFN-I by interacting with MAVS (74, 75). Based on the above research, S (S1 and S2 subunit), N (NTD and CTD domain), E and M proteins are all have great potential to become targets for the development of anti-SARS-CoV-2 drugs and vaccines (**Figure 4**).

### Virulence Factor Targets

Virulence factors (VFs) are molecules with virulence properties such as invasiveness and toxins produced by the metabolism of viruses and bacteria, which mainly inhibit or evade the host’s immune response when infecting the host and obtain nutrients from the host for self-proliferation (76). At present, little is known about the virulence factors of SARS-CoV-2, and there are three virulence factors, namely nsp1, nsp3c and ORF7a, which are considered to be most likely involved in interfering with the innate immunity of the host to assist in immune escape of the virus (77–79). Specifically, nsp1 induces the degradation of mRNA and inhibits the production of IFN-I by interacting with host cell 40S ribosomal subunits, while nsp3c combines with host cell ADP-ribose to resist innate immunity (77, 80). In addition, ORF7a of SARS-CoV-2 directly binds to bone marrow stromal antigen 2 (BST-2), which reduces its activity by blocking the glycosylation of BST-2 and ultimately inhibits the release of the assembled virus (79). In view of the high feasibility of virulence factors as potential targets for SARS-CoV-2 research, the development of drugs that affect the production and effects of virulence factors will be another important clue to explore the fight against SARS-CoV-2-induced COVID-19.

### Hose Specific Receptor or Enzyme Targets

Authoritative studies have confirmed that host cell ACE2 is the specific receptor to which the SARS-CoV S protein receptor binding domain (RBD) binds. The latest research has found that the host receptors of SARS-CoV-2 and SARS-CoV have a high degree of consistency, which indicates that there is also an important interaction between the spike RBD of SARS-CoV-2 and ACE2 (81). During the infection stage, the RBD of the S protein S1 subunit recognizes and binds to the cell surface ACE2 receptor, which promotes the weakening or disappearance of the interaction between S1 and the S2 subunit, thereby exposing the S2 subunit (82). Subsequently, the S2 subunit changes conformation by inserting the fusion peptide (FP) into the host cell membrane, resulting in the formation of a six-helix bundle (6HB) between HR1 and HR2, which ultimately promotes fusion of the viral membrane with the host cell membrane (83). According to the receptor binding motif (RBM) analysis, a large number of amino acid residues necessary for binding to ACE2 are completely retained in the S protein of SARS-CoV-2, which is consistent with the previous discovery that the virus uses ACE2 to enter the host cell (84). Based on a number of authoritative studies, ACE2 will be the most valuable host cell target in preventing the entry and infection of SARS-CoV-2.

In addition, TMPRSS2 can cut off the spike to trigger SARS- and MERS-CoV infection. In a study of SARS-CoV-2, it was found that the virus uses TMPRSS2 instead of cathepsin B and L (CatB/L) to activate the S protein, and the spreading process may also be closely related to the activity of TMPRSS2 (85). Another study found that TMPRSS2 inhibitors can significantly inhibit the SARS-CoV-2 spike protein from entering a cell line expressing TMPRSS2, while promoting the expression of TMPRSS2 can cancel this inhibitory effect, which indicates that the initiation of the SARS-CoV-2 spike protein is dependent on TMPRSS2 (86). Furthermore, an *in vitro* study showed that camostat mesylate, a serine protease inhibitor, can potently stop the virus from entering Caco-2 (TMPRSS2^+^) cells rather than 293T (TMPRSS2^-^) cells by inhibiting the activity of TMPRSS2 (87). The above results suggest that inhibiting TMPRSS2 to treat patients with SARS-CoV-2 infection will be a promising and valuable therapeutic strategy.

CD147 is a highly glycosylated single-pass transmembrane glycoprotein that has been found to play an indelible role in tumor development, plasmodium invasion, virus infection and other processes (88). During SARS-CoV invasion of host cells, CD147 molecules can interact with cyclophilin A (CyPA) to mediate a similar mechanism of action in HIV-1 invasion, while the CD147 antagonist peptide (AP)-9 can strongly bind to HEK293 cells and exert its anti-SARS-CoV effect (89). In view of the high similarity between SARS-CoV and SARS-CoV-2, some studies have attempted to explore the possible role of CD147 in host cell invasion by SARS-CoV-2 (90). The results show that blocking host cell CD147 can significantly inhibit SARS-CoV-2 infection, suggesting that CD147 is likely to be another potential surface receptor independent of ACE2 (91). A study used the humanized anti-CD147 monoclonal antibody called meplazeumab (60 μg/ml), which can prevent virus invasion and the subsequent inflammation caused by SARS-CoV-2 and its variants, including variants α, β, γ and δ, with inhibition rates of 68.7, 75.7, 52.1, 52.1 and 62.3%, respectively (92). Furthermore, CD147 genetically modified mice are more sensitive to SARS-CoV-2 and variants such as α and β, causing the same pathological changes as COVID-19 (93). In addition, surface plasmon resonance analysis confirmed that there is an interaction between CD147 and the S protein (90). This evidence indicates that SARS-CoV-2 can also enter host cells by binding to the CD147 receptor. However, the question of whether CD147 is a coreceptor, a secondary receptor or a completely independent new receptor still needs more research to be verified. However, CD147 is a novel potential therapeutic target with further exploration value in research on fighting SARS-CoV-2 infection. While researchers have multiplied their hopes for discovering this new infection mechanism, several studies have suggested that there is no direct interaction between RBD and CD147, raising doubts about its role as a coreceptor and potential as a therapeutic target (94, 95). Science has always been developed through constant questioning. The conflicting results do not discourage us but instead provide us with new research clues. In any case, more research needs to be done to strengthen the reliability of this finding.

SARS-CoV-2 infection mainly relies on the interaction of the viral surface S protein and the well-known host cell surface receptor ACE2. However, the low expression of ACE2 in the respiratory system makes it difficult to fully explain why SARS-CoV-2 mainly infects the human respiratory system. Along with the continuous deepening of exploration, researchers proved that the AXL protein on lung cells can bind to the spike protein and show a relatively obvious colocalization phenomenon on the cell membrane through large-scale screening and a series of biochemical cellular experiments (96). Interestingly, AXL does not bind to the RBD of the S protein but instead binds to the NTD region at the N-terminus. Meanwhile, a study also found that AXL has significant retention in almost all types of airway cells, including type I/II lung epithelial cells, fibroblasts, basal cells, endothelial cells, smooth muscle cells and myeloid cells. In addition, overexpression of AXL can effectively promote the invasion of SARS-CoV-2, while knocking out AXL in human lung epithelial cells significantly reduces SARS-CoV-2 infection (97). At the same time, clinical data from patients with SARS-CoV-2 also show that the expression level of AXL is highly correlated with severe infections (98). The use of soluble AXL protein can effectively antagonize SARS-CoV-2 infection of lung cells, suggesting that AXL is another potential target during SARS-CoV-2 infection, and targeted or AXL-based drugs may be used for future clinical interventions against SARS-CoV-2 infection.

## Potential Therapeutic Strategies and Promising Anti-SARS-CoV-2 Drugs

### Small Molecule Inhibitors

Drawing lessons from the research and development experience of SARS-CoV and MERS therapeutic drugs and the current authoritative research about SARS-CoV-2, we need to explore small molecule inhibitors that can prevent the novel coronavirus and its epidemic from two directions (99): 1. This type of inhibitor targets viral proteins, such as the S protein, viral enzymes (PL^pro^, 3CL^pro^, RdRP and helicase) and some important structural proteins; 2. This type of inhibitor interacts with host cell surface proteins, such as receptor (ACE2 or AXL) or coreceptor (heparin sulfate), serine protease TMPRSS2, etc., to block virus invasion and some signal regulators of the human immune system, as shown in **Figure 4**. At the same time, the corresponding development strategies are mainly divided into three categories: 1. Virtual screening: High-throughput screening is carried out to identify possible lead compounds from existing compound databases, such as ZINC, DrugBank, or ChemDiv, on the basis of structural biology and homology modeling analysis of protein structure. 2. Experimental high-throughput screening (HTS): Identify small molecules in the active compound library, including approved drugs, clinical trial candidates, and even internal compound databases. 3. Reposition the application of clinical and preclinical drugs (100, 101). That is the so-called “new use of old medicine”. In addition, the computer-aided design and fragment-based drug exploration are also important strategies.

Under the guidance of these strategies, a variety of small molecule inhibitors targeting different stages of the SARS-CoV-2 life cycle have been discovered. When trying to block SARS-CoV-2 entry by targeting the S protein, the researchers found that Arbidol, Bictegravir, Dolutegravir, and Tizoxanide all have such a conformation that they can bind to the key sites of the S protein with a very high binding energy (102). Arbidol mainly binds to the S1 and S2 subunits of SARS-CoV-2 to promote tight subunit binding, which not only prevents the S1 subunit from falling off, but also impedes the membrane fusion function of the S2 subunit, eventually preventing virus entry. *In vitro* experiments showed that Arbidol has satisfactory activity against SARS-CoV-2 with IC_50_ and 50% cytotoxic concentration (CC_50_) values of 4.11 and 31.79 μmol/L, respectively, and the selectivity index (SI) was 7.73 (103). Bictegravir and Dolutegravir combine between the RBD and NTD of two adjacent S1 monomers, which can prevent SARS-CoV-2 entry by restricting the interaction between the spike RBD and ACE2 receptor (104). In addition, Tizoxanide not only affects the stability of the S1 subunit through hydrogen bonds and van der Waals forces to prevent the RBD in the metastable conformation of the S1 subunit from binding to ACE2 but also affects the membrane fusion of the S2 subunit and host cell (105). Importantly, structural optimization of these molecules produces 9 new small molecules with better anti-SARS-CoV-2 activity, which provides important references for the discovery, development and optimization of small molecule inhibitors targeting the S protein (102). According to the research experience of SARS/MERS-CoV, the design of viral fusion interference peptides based on the properties of heptad repeat 1 (HR1) and heptad repeat 2 (HR2) of the S2 subunit is also an important strategy for the research of small molecule inhibitors of SARS-CoV-2. A pancoronavirus fusion inhibitor peptide, EK1, was designed to inhibit a variety of CoVs and inhibit SARS-CoV-2 S protein-mediated membrane fusion and pseudovirus infection in a dose-dependent manner. Subsequently, its improved version, lipopeptide EK1C4 was designed to have the same inhibitory effect at IC_50 values_ of 1.3 and 15.8 nmol/L, and these two results were 241- and 149-fold those of the former, respectively (106). In addition, another lipopeptide, IPB02, designed based on the HR2 sequence also showed a similar effect (107). Furthermore, SARS-CoV-2-HR2P, a peptide directly based on the amino acid sequence of SARS-CoV-2 HR2, showed a potent membrane fusion inhibition with an IC_50_ of 0.18 mmol/L (106). Unlike SARS-CoV-2-HRP2, which is designed on a single amino acid, [SARSHRC-PEG4]2-chol, as a dimeric lipopeptide has better membrane fusion inhibition and lower cytotoxicity against SARS-CoV-2 entry (108). After that, one study designed a peptide SBP1 composed of 23-mer peptides to prevent the virus from entering the host cell by disrupting the combination of SARS-CoV-2-RBD and ACE2 (109). To inhibit the combination of viral S protein and ACE2, a study designed two types of peptide inhibitors, AHB1/2 and LCB1/3, by two *de novo* synthesis approaches around the ACE2 helix structure and RBD motif, which have a strong SARS-CoV-2 neutralization effect with IC_50 values_ of 35/15.5 nmol/L and 23.54/48.1 pmol/L, respectively (110). A study identified a fibronectin-derived anticancer peptide ATN-161 from existing peptides that can prevent the binding of the S protein to ACE2, thereby reducing SARS-CoV-2 infection with an IC_50_ of 3.16 mmol/L (111). In light of this, the design of small molecule inhibitors for the S protein should focus on the protein structure, amino acid sequence and motif characteristics of the RBD, S1 and S2 subunits. When targeting the host cell ACE2 receptor, it has recently been suggested that ACE2 inhibitors, such as captopril and enalapril, may be effective for those who have experienced SARS-CoV-2-induced pneumonia (112). Nicotinamide analogs, such as nicotinamide riboside (NR) and nicotinamide mononucleotide (NMN), are an important class of natural vitamin derivatives. A relevant study found that it can effectively inhibit ACE2, so it is considered a potential inhibitor for the treatment of COVID-19 (113). However, these are only theoretical speculations and almost no basic or clinical research verification. It is possible that such suggestions will gradually fade out of people’s field of vision. At present, the development of anti-SARS-CoV-2 drugs targeting ACE2 is mainly focused on peptides, antibodies and other biochemical products, including ACE2 antibody, ACE2-scFv, ACE2 nanobody, and ACE2-Fc (114, 115). Although TMPRSS2 is the gateway for SARS-CoV-2 host cells to enter, there have not been many breakthroughs in the research of small molecule inhibitors against this target. The known TMPRSS2 inhibitor camostat in a clinical trial against COVID-19 shows excellent abilities to reduce the death risk and hospital stay (87). Recently, a study demonstrated that the camostat-like drug nafamostat mesylate can prevent the SARS-CoV-2 membrane fusion caused by TMPRSS2 at a concentration of its less than one tenth, suggesting that nafamostat mesylate may be a promising inhibitor against SARS-CoV-2 infection by targeting TMPRSS2 (116). Other studies identified a variety of serine protease experimental inhibitors (DB03782, DB03213, and DB04107) and potential molecules (Z126202570, Z46489368, and Z422255982) through homology modeling and molecular docking/dynamic simulation and embraced binding free energy calculations that may effectively inhibit the TMPRSS2, which all contain a positively charged warhead similar to nafamostat and camostat (117). However, these molecules need to be determined by in-depth mechanistic research. A recent study discovered a covalent small molecule ketobenzothiazole (kbt) serine protease inhibitor, MM3122, whose structure is completely different from camostat and nafamostat and is said to be effective (86). All these results indicate that the study of small molecule inhibitors targeting TMPRSS2 for SARS-CoV-2 will be a good choice. In addition to the ACE2-mediated virus entry pathway, CD147-mediated viral entry is likely to become the second pathway of SARS-CoV-2 invasion. Although still controversial, this does not affect the research of drugs targeting CD147 in the prevention of SARS-CoV-2 infection (90). At present, the drugs targeting CD147 are mainly monoclonal antibodies, and research on small molecule compounds is rarely involved, which will be a breakthrough in future research. The latest research suggests that AXL is a candidate receptor for SARS-CoV-2, which can promote the infection of lung and bronchial epithelial cells (97). As a receptor tyrosine kinase (RTK), there is currently no report on the use of small molecule compounds targeting AXL for the treatment of SARS-CoV-2 infection, but we can refer to the research of RTK small molecule inhibitors in tumors to discover potential small molecule inhibitors of AXL for preventing the entry of SARS-CoV-2.

When considering inhibiting SARS-CoV-2 replication, the study found a variety of promising small molecule inhibitors. 3CL^pro^ (nsp5) is one of the most ideal targets for discovering inhibitors of SARS-CoV-2. The study found that the amino Cys145 residue in the catalytic pocket of 3CL^pro^ is an effective target for exploring small molecular covalent inhibitors of SARS-CoV-2 and other coronaviruses (118). A fluorescence resonance energy transfer study found that the 8-aminoquinoline antimalarial drug tafenoquine can induce the transformation of 3CL^pro^ to expose the hydrophobic pocket and promote the protein aggregation, ultimately reducing the activity of 3CL^pro^ and repressing SARS-CoV-2 RNA replication with an IC_50_ near 2.5 μmol/L, which is appropriately 1/4 that of hydroxychloroquine (119). Although Lopinavir-Ritonavir (*Kaletra*) was initially used in the treatment of SARS-CoV-2 because of its ability to block the replication of SARS-CoV and MERS by inhibiting 3CL^pro^, the latest research results do not support its use in the treatment of COVID-19 (120). A study screened FDA-approved drug libraries and found that the anticoagulant dipyridamole (DIP) may bind to 3CL^pro^ to inhibit more than 50% of SARS-CoV-2 replication in Vero E6 cells at 100 nmol/L. After 2 weeks of DIP treatment, 8 critically ill patients improved significantly (121). With the continuous understanding of the structure of the 3CL^pro^ protein, more small molecule inhibitors have been discovered, and some of them have been in clinical trials. Compounds 11a and 11b have been screened and confirmed to have a strong inhibitory effect on SARS-CoV-2 3CL^pro^, with IC_50 values_ of 0.053 μmol/L and 0.040 μmol/L, respectively; EC_50 values_ of 0.53 μmol/L and 0.72 μmol/L, respectively; and have good pharmacokinetic properties (122). At present, 11a (DC402234) has submitted a clinical application registration declaration and has obtained FDA conditional clinical trial approval (Phase I: NCT04766931). After screening, the *in vivo* antiviral test results of the small molecule compounds MI-09 and MI-30 showed that oral or intraperitoneal injection of these compounds can significantly reduce the lung viral load and lung pathological damage in a SARS-CoV-2-infected transgenic mouse model (123). Although various research results and different inhibitors of 3CL^pro^ have been shown in front of people one after another, the clinical entry is extremely limited; only the four inhibitors (PF-07304814 [phase III: NCT04501978] and PF-07321332 [phase III: NCT05047601] developed by Pfizer, USA; the aforementioned 11a (DC402234 made by Frontiers, China [phase I: NCT04766931]; and the code-named S-217622 produced by Shionogi Inc., Japan [phase II/III: jRCT2031210350) are in clinical trial (124). PL^pro^ (nsp3) has also received much attention due to its important role in the replication and invasion of SARS-CoV-2. Some noncovalent small molecule inhibitors (rac3j, rac3k and rac5c) that have been effective against SARS-CoV can target SARS-CoV-2 PL^pro^ to prevent the self-processing of nsp3 in cells, thus reducing viral-induced CPE at high concentrations (33 μmol/L) (125). Based on the crystal structure of SARS-CoV-2 PL^pro^, researchers obtained useful data from the FDA-approved drug database and identified 147 potential inhibitors of SARS-CoV-2 PL^pro^. In Vero E6 cells, dronedarone, an ion channel modifier, has good antiviral activity against SARS-CoV-2-induced CPE with an IC_50_ of 4.5 μmol/L (CC_50_ of 12.1 μmol/L) (126). The naphthalene-based inhibitor, GRL-0617, can effectively inhibit the activity of SARS-CoV-2 PL^pro^ with an IC_50_ of 2.2 μmol/L, and its mechanism is not limited to occupying the substrate pocket but expands to seal the substrate binding entrance cleft, thereby preventing the binding of the substrate (62). At present, the crystal structure of PL^pro^ has been completely resolved (PDB code: 6W9C), and more small molecule compounds will be discovered as the crystal structure is fully analyzed. To date, no small molecule inhibitors against PL^pro^ have entered clinical studies, which suggests that there is still a long way to go in the development of PL^pro^-targeted small molecule inhibitors against SARS-CoV-2. RdRP (nsp 12) has become an important target for the development of anti-SARS-CoV-2 drugs because it participates in the virus replication process as a key enzyme that catalyzes the synthesis of the SARS-CoV-2 genome. A research group from Shanghai, China, successfully analyzed the three-dimensional structure of the RdRP-nsp7-nsp8 complex at near-atomic resolution (with an overall resolution of 2.9 Å) using cryo-electron microscopy, which lays a solid foundation for the design of antiviral inhibitors based on the RdRP structure (127). As research on SARS-CoV-2 RdRP continues, multiple potential drugs have been discovered and confirmed. Remdesivir [GS-5734], a nucleotide analog originally used to fight Ebola virus, was first proposed for the treatment of COVID-19 patients because it can be used as a substrate for the RdRP. In Vero E6 cells at a SARS-CoV-2 MOI of 0.05, remdesivir shows an ideal potential to fight SARS-CoV-2 with IC_50_ = 0.77 μmol/L, CC_50_ > 100 μmol/L and SI > 129.87, which also quickly promotes the quick access of RdRP small molecule inhibitors to global phase III clinical trials and their direct use in some regions (128). However, things are always dramatic. The latest clinical trial results published by the WHO do not seem to be optimistic about this small molecule inhibitor (129). As far as COVID-19 hospitalized patients are concerned, it has little or no impact on indicators such as overall mortality and duration of hospital stay. Regardless of the outcome, the emergence of remdesivir has provided an important reference and motivation for the research of small molecule inhibitors targeting RdRP. A subsequent study screened a century-old classic drug, suramin, and a variety of derivatives, which exhibited a more than 20-fold ability to fight SARS-CoV-2 infection with remdesivir by targeting RdRP (66). Another small-molecule inhibitor called favipiravir (T-705) targets RdRP to mildly resist SARS-CoV-2 infection with an IC_50_ of 61.88 μmol/L, CC_50_ > 400 μmol/L and SI > 6.46 (130). Several clinical trials (ChiCTR2000029600/200030254, etc.) have shown that favipiravir may accelerate virus clearance and alleviate the progression of COVID-19, which lays a solid foundation for its clinical application and provides a structural basis and strong evidence for the development of broad-spectrum antiviral drugs based on the strategy of “old drugs and new use”. A study reported that the oral broad-spectrum ribonucleoside analog β-D-N4-hydroxycytidine [EIDD-1931] showed good anti-SARS-CoV-2 activity in Vero cells with an IC_50_ of 0.3 μmol/L (131). In addition, the oral EIDD-1931 prodrug molnupiravir (MK-4482, EIDD-2801, Merck Sharp & Dohme Corp, USA), due to its ideal anti-coronavirus effect, has ended phase III clinical trials (NCT04575584, NCT04575597 and NCT04939428) ahead of schedule and is expected to be launched in the United States soon (131). The oral purine nucleotide prodrug AT-527 developed by Roche is expected to have a good phase III clinical trial (NCT04889040) result (132). These studies provide hopes and directions for the development of small molecule inhibitors targeting RdRP during SARS-CoV-2 infection. At present, there are already several small molecule inhibitors that target helicases, such as bananins, 5-hydroxychromone derivatives, and SSYA10-001, which are expected to be used in SARS-CoV-2-related experiments (133). In addition, authoritative studies suggest that the classic old drug clofazimine has the ability to inhibit the helicase activity of SARS-CoV-2, suggesting that it may play a role in controlling the current COVID-19 pandemic and the emergence of CoVs in the future (134). Although there has been hope, the greatest challenge is the relatively low selectivity of small molecule inhibitors targeting helicase, and there is no drug targeting helicase that exceeds preclinical development. However, the development of small molecule helicase inhibitors may provide another effective treatment option for the COVID-19 pandemic.

Studies are also concerned that several small molecule inhibitors can fight SARS-CoV-2 *via* immunoregulatory and inflammatory functions, and the specific details are introduced in the “Significant Symptomatic Therapeutic Agents” section.

At present, the continuous rapid screening of small molecule databases based on SARS-CoV-2 potential targets has found some effective lead compounds or candidate drugs, which will promote the continuation of basic research and clinical trials of small molecule inhibitors for COVID-19 (**Table 2**). In addition, computer-based drug design is icing on the cake for accelerating the screening and development of small molecule inhibitors, but it is conservatively estimated that new targeted interventions will still take some time. Considering the current spread of the novel coronavirus disease and the continuing case fatality rate, rapid screening of FDA-approved and clinical trial drugs is a more practical method because “old drugs and new use” may reduce development costs and shorten development time (232). To date, a large number of small molecule inhibitors against SARS-CoV-2 infection have been screened; however, many of these studies have not been fully implemented (233). Meanwhile, the safety of some confirmed promising anti-SARS-CoV-2 small molecule inhibitors or drugs is also unknown, especially reproductive toxicity, which imposes more difficulties on the clinical translation of small molecule inhibitors. Therefore, adequate research needs to be carried out to maximize safety and avoid false positive effects. The mutation of the virus and the SARS-CoV-2 epidemic have made the discovery of vaccines and drugs more uncertain. In the long run, there is still much work to be done in the screening, validation, clinical research and clinical application of specific or broad-spectrum small molecule inhibitors for SARS-CoV-2 virus entry, replication, or prevention.

**Table 2:** List of drugs that may be effective in the preclinical and clinical phases for COVID-19.

Drug Name	Drug Type	Target	Study Phase	Test Effect	Reference Doi
Arbidol	Small molecule compound	S1/S2 subunit of Spike protein	Phase IV (NCT04252885; NCT04260594)	Prevents S1 subunit from falling off and membrane fusion of S2 subunit, and SARS-CoV-2 entry	Wang et al. (103)
Bictegravir	Small molecule compound	S2 subunit of Spike protein	Preclinical	Prevents the SARS-CoV-2 entry by restricting the interaction between Spike RBD and ACE2	Sun et al. (102)
Dolutegravir	Small molecule compound	S2 subunit of Spike protein	Preclinical	Prevents the SARS-CoV-2 entry by restricting the interaction between Spike RBD and ACE2	Sun et al. (102)
Tizoxanide	Small molecule compound	S1/S2 subunit of Spike protein	Preclinical	Prevents the S1 subunit from binding to ACE2, and S2 subunit membrane fusion	Sun et al. (102)
EK1	Peptide	Spike protein HR2	Preclinical	Against Spike protein-mediated membrane fusion and pseudovirus infection	Xia et al. (135)
EK1C4	Lipopeptide	Spike protein HR2	Preclinical	Against Spike protein-mediated membrane fusion and pseudovirus infection	Xia et al. (106)
IPB02	Lipopeptide	Spike protein HR2	Preclinical	Against Spike protein-mediated celle-cell fusion and pseudovirus infection	Zhu et al. (107)
SARS-CoV-2-HR2P	Peptide	Spike protein HR2	Preclinical	Against Spike protein-mediated membrane fusion and pseudovirus infection	Xia et al. (106)
[SARS_HRC_-PEG_4_]_2_-chol	Dimeric lipopeptide	Spike protein HR2	Preclinical	Against Spike protein-mediated membrane fusion and SARS-CoV-2 entry	de Vries et al. (108)
SBP1	23-mer peptide fragment	SARS-CoV-2-RBD	Preclinical	Block the interaction between Spike protein and ACE2, and SARS-CoV-2 entry	Ucar et al. (109)
AHB1/3	Peptide	SARS-CoV-2-RBD	Preclinical	Inhibit the SARS-CoV-2 attachment between Spike protein and ACE2, and viral neutralization	Cao et al. (110)
LCB1/3	Peptide	SARS-CoV-2-RBD	Preclinical	Inhibit the SARS-CoV-2 attachment between Spike protein and ACE2, and viral neutralization	Cao et al. (110)
ATN-161	Integrin binding peptide	Spike protein, ACE2	Preclinical	Inhibit SARS-CoV-2 attachment through α5β1 integrin-based mechanism	Beddingfield et al. (111)
Captopril	ACE inhibitor	ACE2	Phase II (NCT04355429)	Inhibit the interaction between Spike protein and ACE2, and viral neutralization	Milne et al. (136)
Enalapril	ACE inhibitor	ACE2	Phase III (NCT04591210)	Inhibit the interaction between Spike protein and ACE2, and viral neutralization	Bauer, et al. (112)
Camostat mesylate	Serine protease inhibitor	TMPRSS2	Phase II (NCT04455815)	Blocks the TMPRSS2 activity induced Spike protein priming and SARS-CoV-2 entry	Hoffmann et al. (137)
Nafamostat mesylate	Serine protease inhibitor	TMPRSS2	Phase II/III (NCT04455815)	Blocks the TMPRSS2 activity induced Spike protein priming and SARS-CoV-2 entry	Hempel et al. (138)
Z126202570; Z46489368; Z422255982	Serine protease inhibitor	TMPRSS2	Preclinical	Blocks the TMPRSS2 activity induced Spike protein priming and SARS-CoV-2 entry	Alzain and Elbadwi, et al. (117)
MM3122	Serine protease inhibitor	TMPRSS2	Preclinical	Blocks the TMPRSS2 activity induced Spike protein priming and SARS-CoV-2 entry	Mahoney et al. (86)
Tafenoquine	8-aminoquinoline antimalarial drug	3CL^pro^	Preclinical	Induces the transformation of 3CL^pro^ conception, inhibits the activity of 3CL^pro^ and represses the SARS-CoV-2 RNA replication	Achutha et al. (119)
Dipyridamole (DIP)	Anticoagulant	3CL^pro^	Preclinical	Inhibits the activity of 3CL^pro^ and represses the SARS-CoV-2 RNA replication	Liu et al. (121)
Compound 11a	Pseudopeptide lead compound	3CL^pro^	Phase I (NCT04766931)	Inhibits the activity of 3CL^pro^ and represses the SARS-CoV-2 RNA replication	Dai et al. (122)
Compound 11b	Pseudopeptide lead compound	3CL^pro^	Preclinical	Inhibits the activity of 3CL^pro^ and represses the SARS-CoV-2 RNA replication	Dai et al. (122)
MI-09	Boceprevir or telaprevir derivatives	3CL^pro^	Preclinical	Inhibits the activity of 3CL^pro^ and represses the SARS-CoV-2 RNA replication	Qiao et al. (123)
MI-30	Boceprevir or telaprevir derivatives	3CL^pro^	Preclinical	Inhibits the activity of 3CL^pro^ and represses the SARS-CoV-2 RNA replication	Qiao et al. (123)
PF-07304814	Phosphate prodrug of PF-00835231	3CL^pro^	Phase I (NCT04535167)	Inhibits the activity of 3CL^pro^ and represses the SARS-CoV-2 RNA replication	Yap et al. (139)
PF-07321332	Orally active pseudopeptide 3CL^pro^ inhibitor	3CL^pro^	Phase III (NCT04960202)	Inhibits the activity of 3CL^pro^ and represses the SARS-CoV-2 RNA replication	Zhao et al. (140)
S-217622	Orally active reversible covalent 3CL^pro^ inhibitor	3CL^pro^	Phase II/III (jRCT2031210350)	Inhibits the activity of 3CL^pro^ and represses the SARS-CoV-2 RNA replication	https://www.shionogi.com/jp/ja/news/2021/10/211021_2.html
GRL-0617	Naphthalene-based selective noncovalent PL^pro^ inhibitor	PL^pro^	Preclinical	Inhibits of PL^pro^ to impair the SARS-CoV-2-induced cytopathogenic effect, maintain the antiviral interferon pathway and reduce viral replication	Pitsillou et al. (141) Shin et al. (62)
Remdesivir	Monophosphoramidate prodrug of adenosine analogue	RdRP	Phase II/III (NCT04431453)	Blocks the RdRP activity to block the SARS-CoV-2 replication and infection, thus reducing the time to recovery in COVID-19 patients	Kokic et al. (142)
Suramin	Non-nucleoside RdRP inhibitor	RdRP	Phase II (ChiCTR2000030029)	Inhibits the RdRP activity to block the SARS-CoV-2 replication and infection	Yin et al. (143)
Favipiravir	Nucleotide analogue	RdRP	Phase III (NCT04558463)	Inhibits the RdRP activity to block the SARS-CoV-2 replication and infection	Naydenova et al. (144) Ninove et al. (145)
EIDD-1931	Ribonucleoside analogue	RdRP	Phase I/II (NCT04746183)	Prevents the synthesis of RdRP and promotes the mutation of SARS-CoV-2 RNA bases to kill the virus, reduce the viral load and finally clear the infection	Miller et al. (146) Jena et al. (147)
EIDD-2801 (molnupiravir)	Oral EIDD-1931 prodrug (ribonucleoside analogue)	RdRP	Phase II (NCT04405570)	Anti-SARS-CoV-2 after being metabolized into EIDD-1931 in the body	Wölfel et al. (148) Sheahan et al. (131)
AT-527	Double Prodrug of a Guanosine Nucleotide Analog	RdRP	Phase III (NCT04889040)	It selectively inhibits the RdRP activity to block the SARS-CoV-2 replication and infection	Good et al. (132)
Bananins	Drug-like compound	Helicase	Preclinical	Blocks the virus replication and load by inhibiting the helicase activity	Spratt et al. (133)
SSYA10-001	Drug-like compound	Helicase (nsp13)	Preclinical	Blocks the virus replication and load by inhibiting the helicase activity	Spratt et al. (133)
Clofazimine	Anti-tuberculosis drug	Helicase Spike protein	Phase II (NCT04465695)	Inhibits the spike-dependent entry, reduces viral load by disrupting the helicase induced virus replication, and also prevents cytokine storm associated with viral infection	Yuan et al. (134)
BBIBP-CorV	Inactivated (Vero cells) vaccine	Spike protein	Phase III (NCT04993560); Approved for emergency utilization worldwide	Elicits high levels of neutralizing antibodies (anti-receptor-binding domain (RBD) IgG, as well as anti-spike protein (S) IgG and IgA antibodies) and T cell-mediated immune responses	Wang (149) Xia et al. (150)
CoronaVac	Inactivated (Vero cells) vaccine	S1 domain and RBD of Spike protein	Phase III (NCT05077176); Approved for emergency utilization worldwide	Elicits the development of humoral immunity against SARS-CoV-2, particularly Ig anti-RBD	Zhang et al. (151) Vacharathit et al. (152)
WIBP vaccine	Inactivated (Vero cells) vaccine	Spike protein	Phase III (NCT04510207)	Elicits high levels of neutralizing antibodies and T cell-mediated immune responses	Al et al. (153)
BBV152 (Covaxin)	Whole-virion inactivated (Vero cells) vaccine	Spike protein	Phase III (NCT04641481)	Induces high titres of specific IgG and neutralizing antibodies and enhances cytokine and chemokine responses	Ella et al. (154) Ella et al. (155)
ChAdOx1 nCoV-19/AZD1222	Non-replicating adenovirus vectored vaccine	Spike protein	Phase III (NCT05059106)	Induces high anti-spike neutralizing antibody titers, as well as Fc-mediated functional antibody responses	Voysey et al. (156) Ramasamy et al. (157)
Ad26.COV2.S	Non-replicating adenovirus 26 vectored vaccine	Spike protein	Phase III (NCT04505722)	Induces high titres and stable neutralizing antibodies and enhances T-cell responses	Sadoff et al. (158)
Ad5-nCoV	Non-replicating adenovirus type 5 vectored vaccine	Spike protein	Phase III (NCT04540419); Approved for emergency utilization in China	It generates S1 IgG antibodies to induce strong humoral and cellular immune responses	Guzmán-Martínez et al. (159) Wu et al. (160)
Gam-COVID-Vac	Non-replicating adenovirus 5 and 26 vectored vaccine	Spike protein	Phase III (NCT04642339) Approved for emergency utilization in Russia	Induces high titres of specific IgG and neutralizing antibodies and enhances T-cell responses	González et al. (161) Logunov et al. (162)
GRAd-COV2	Non-replicating defective Simian adenovirus vectored vaccine	Spike protein	Phase II/III (NCT04791423)	Elicits both functional antibodies that neutralize SARS-CoV-2 infection and block Spike protein binding to the ACE2 receptor, and a robust, T helper (Th)1 dominated cellular response	Lanini et al. (163)
VXA-CoV2-1	Non-replicating adenovirus Ad5 vectored vaccine	Spike protein	Phase I (NCT04563702)	Induces anti-spike IgG and neutralizing antibodies with the sera demonstrating neutralizing activity	Johnson et al. (164)
hAd5-S-Fusion+N-ETSD	Non-replicating adenovirus Ad5 vectored vaccine	Spike protein N protein	Phase I/II (NCT04845191)	Induces neutralizing antibodies and Th1-prone N- and S-specific T-cell responses, providing complete protection of the nasal cavity and lungs against SARS-CoV-2 infection	Gabitzsch et al. (165)
LV-SMENP-DC	Minigenes engineered based on multiple viral genes, lentiviral vectored (NHP/TYF) modified dendritic cell vaccine	Spike protein	Phase I/II (NCT04276896)	Induces neutralizing antibodies and T-cell responses	Mahrosh et al. (166)
Pathogen-specific aAPC	Minigenes engineered based on multiple viral genes, lentiviral vectored (NHP/TYF) vaccine	Antigen presenting cells	Phase I (NCT04299724)	Induces neutralizing antibodies and T-cell responses	Mahrosh et al. (166)
DelNS1-2019-nCoV-RBD-OPT1	Replicating intranasal based-RBD flu vectored vaccine	Spike protein	Phase II/III (ChiCTR2100048316/ChiCTR2100051391)	Simulates the natural infection pathway of respiratory viruses to activate local and systemic T-cell immune response to prevent the SARS-CoV-2 infection	Wang et al. (167)
VSV-ΔG-SARS-CoV-2-S/IIBR-100	Replicating viral VSV vectored vaccine	Spike protein	Phase II/III (NCT04990466)	It develops spike-specific antibodies in antisera to prevent the SARS-CoV-2 infection	Yahalom-Ronen et al. (168)
TMV-083/V-591	Attenuated measles-vector based vaccine	Spike protein	Phase I/II (NCT04497298/NCT04498247); Stop R&D	Increases the geometric mean titers (GMTs) of anti-SARS-CoV-2 Spike protein serum neutralizing antibody to prevent the SARS-CoV-2 infection	Scarabel, Lucia, et al. (169)
V590	Recombinant VSV-vector based vaccine	Spike protein	Phase I (NCT04569786); Stop R&D	Increases the geometric mean titers (GMTs) of anti-SARS-CoV-2 Spike protein serum neutralizing antibody	Scarabel et al. (169)
MVA-SARS-2-S	Nonreplicating modified vaccinia virus Ankara vectored vaccine	Spike protein	Phase I (NCT04569383)	The robust expression of Spike protein as antigen to produce S-specific CD8+ T cells and serum antibodies binding to Spike protein that neutralized SARS-CoV-2.	Tscherne et al. (170)
ZyCoV-D	DNA vaccine	Spike protein	Phase I/II (CTRI/2020/07/02635); Approved for clinical use in India	It encodes and translate the SARS-CoV-2 Spike protein, which stimulates the host to produce high titres of virus-neutralizing antibodies and robust T cell immune response, thereby completely blocking the virus entry and subsequent infection	Momin et al. (171) Dey et al. (172)
INO-4800	DNA vaccine	Spike protein	Phase III (NCT04642638)	It induces antibodies to block SARS-CoV-2 Spike protein binding to the host receptor ACE2 and produces high titres of virus-neutralizing antibodies and robust cell immune response, thereby completely blocking the virus entry and subsequent infection	Tebas et al. (173) Smith et al. (174)
BNT162b2	Nucleoside-modified mRNA vaccine	Spike protein	Phase III (NCT04955626); Approved for clinical use	It mimics and encodes the SARS-CoV-2 spike protein, which stimulates the host to produce high titres of virus-neutralizing antibodies and robust T cell immune response, thereby completely blocking the virus entry and subsequent infection	Polack et al. (175) Liu et al. (176)
mRNA-1273	Lipid nanoparticle-encapsulated mRNA vaccine	Spike protein	Phase III (NCT04860297)	It mimics and encodes the SARS-CoV-2 spike protein, which stimulates the host to produce high titres of virus-neutralizing antibodies and robust immune response, thereby completely blocking the virus entry and subsequent infection	Baden et al. (177) Jackson et al. (178)
CVnCoV	Lipid nanoparticle-encapsulated naturally occurring nucleotides mRNA vaccine	Spike protein	Phase III (NCT04860258)	It mimics and encodes the SARS-CoV-2 surface spike protein, which stimulates the host to produce high titres of virus-neutralizing antibodies and robust T-cell responses, thereby completely blocking the virus entry and subsequent infection	Alexandersen et al. (179) Rauch et al. (180)
ARCT-021	Self-replicating mRNA and nanoparticle delivery system vaccine	Spike protein	Phase II (NCT04728347)	It mimics and encodes the virus surface spike protein, which stimulates the host to produce antibodies to activate cell-mediated immunity, thereby completely blocking the entry of SARS-CoV-2 and subsequent infection	Rappaport et al. (181)
LNP-nCoVsaRNA	Self-amplifying mRNA vaccine	Spike protein	Phase I (ISRCTN17072692)	It mimics the virus surface spike protein gene, triggers the virus to produce spike protein, stimulates the host to produce antibodies and completely blocks the entry of SARS-CoV-2 and subsequent infection	Karpiński et al. (182)
ARCoV	Lipid nanoparticle thermostable mRNA-based Vaccine	Spike protein RBD	Phase III (NCT04847102)	It encodes the viral Spike protein RBD to elicit robust neutralizing antibodies against SARS-CoV-2 as well as a Th1-biased cellular response against the viral challenge	Zhang et al. (183)
hrsACE2	Human recombinant soluble ACE2	ACE2	Preclinical	It prevents the interaction between Spike protein and ACE2, reduce early SARS-CoV-2 infections, and effectively inhibit the viral proliferation in human vascular organs and kidney organs	Monteil et al. (115) Abd et al. (184)
LY-CoV555 LY3819253	S protein neutralizing antibody	Spike protein	Phase II/III (NCT04427501)	It high-affinity binds to the Spike protein RBD to inhibit the ACE2 binding and reduce the viral replication in the upper and lower respiratory tract	Chen et al. (185) Yang et al. (186)
BRII-196 BRII-198	S protein neutralizing antibody	Spike protein	Phase III (NCT04501978)	It binds to different highly conserved epitope on the Spike protein to block viral entry and neutralize live SARS-CoV-2 infection	Yang et al. (186)
SCTA01 HB27	S protein neutralizing antibody	Spike protein RBD	Phase II/III (NCT04644185)	It engages the Spike protein RBD to efficiently neutralize SARS-CoV-2 pseudoviruses as well as authentic SARS-CoV-2	Yang et al. (186) Li et al. (187)
NVX-CoV2373	Recombinant nanoparticle spike protein subunit vaccine	Spike protein	Phase II (NCT05112848)	It elicits high titer anti-S IgG that blocks hACE2 receptor binding, neutralize virus infection and antigen-specific-cells, and protects against SARS-CoV-2 challenge	Tian et al. (188) Keech et al. (189)
RBD219-N1C1	Recombinant protein heterologous vaccine	Spike protein RBD	Preclinical	Stimulate SARS-CoV-2 to produce IgG neutralizing antibodies and induce T-cell immunity	Chen et al. (190) Lee et al. (191)
HR2P polypeptide	Peptide-based membrane fusion inhibitor	Spike protein HR2 domain	Preclinical	It can effectively inhibit SARS-CoV-2 replication and the Spike protein-mediated cell-cell fusion for treating the viral infection	Xia et al. (192) Lu et al. (193)
Lianhua Qingwen Capsule	TCM	multiple targets such as Akt1, MAPK1, IL6, HSP90AA1, TNF, and CCL2, et al	Real World Study	The main ingredients can inhibit multiple protein targets such as Akt1, MAPK1, IL6, HSP90AA1, TNF, and CCL2, et al, to reduce the inflammatory storm, tissue damage and help eliminate virus infection	Xia et al. (194) Yan et al. (195)
Qingfei Paidu Decoction	TCM	3CL^pro^, and multiple targets such as CXCR4, ICAM1, CXCL8, CXCL10, IL6, IL2, CCL2, IL1B, IL4, et al	Real World Study	Multiple main ingredients can inhibit the 3CL^pro^ mediated SARS-CoV-2 replication, and invasion, and anti-inflammatory and immune regulation, and repairing body damage	Yang et al. (196) Li et al. (197)
Huoxiang Zhengqi formula	TCM	3CL^pro^, PI3K/Akt	Real World Study	Multiple main ingredients can inhibit the 3CL^pro^ mediated SARS-CoV-2 replication and improve the PI3K/Akt mediated inflammatory cytokine release and inflammatory storm	Du et al. (198)
Xuebijing injection	TCM	3CL^pro^, ACE2	Real World Study	Multiple components combine with 3CLpro and ACE2 to act on targets such as IL6, CCL2, TNF and PTGS2 to reduce SARS-CoV-2 entry inflammation responses and regulate the immune functions	Qin et al. (199) Feng et al. (200)
Jinhua Qinggan Granules	TCM	3CL^pro^, ACE2	Real World Study	Multiple components combine with 3CL^pro^ and ACE2 to act on targets such as PTGS2, HSP90AB1, HSP90AA1, PTGS1, and NCOA2 to shorten the fever time, increase the recovery rate of lymphocytes and white blood cells, and improve related immunological indicators	Zhang et al. (201) Liu et al. (202)
Tanreqing Injection	TCM	3CL^pro^, CD3^+^ T cell	Real World Study	Multiple main ingredients can inhibit the 3CL^pro^ mediated SARS-CoV-2 replication and improve the CD3^+^ T-cell level to enhance immune function	Zhang et al. (203)
Huashi Baidu Decoction	TCM	3CL^pro^, ACE2	Real World Study	Blocks the ACE2 receptor mediated SARS-CoV-2 host cell entry and inhibits the 3CL^pro^-mediated viral replication and infection	Tao et al. (204) Cai et al. (205)
Shufeng Jiedu Capsule	TCM	3CL^pro^, NF-κB	Real World Study	Inhibits the NF-κB signaling pathway and 3CL^pro^ to reduce the SARS-CoV-2 load, cytokine storm, inflammation and regulate immune response	Chen et al. (206) Xia et al. (207)
Xuanfei Baidu Decoction	TCM	NF-κB signaling pathway	Real World Study	Inhibits the NF-κB mediated cytokine storm and blunts the THP-1-derived macrophages pinocytosis	Li et al. (208)
Reduning injection	TCM	Carbonic anhydrases (CAs), matrix metallopeptidases (MMPs) and multiple pathways like PI3K/Akt, MAPK	Real World Study	Inhibits the overexpression of MAPKs, PKC and p65 NF-κB to reduce cytokine storm, inflammation and lung damage	Cao et al. (209) Xu et al. (210) Jia et al. (211)
Shenmai injection	TCM	Bcl2, MAPK3 and IL-6	Real World Study	Immune regulation for COVID-19	Yang et al. (212)
Quercetin	Plant flavonoid active ingredients of TCM	Multiple enzymes including 3CL^pro^, PL^pro^, RDRP, Spike protein and ACE2	Preclinical	Inhibits multiple SARS-CoV-2 enzymes mediated viral replication, attachment and entry and infection	Derosa et al. (213) Pan et al. (214) Saakre et al. (215)
Kaempferol	The main flavonoid polyphenols of kaempferol galanga L	ACE2 and 3CL^pro^	Preclinical	Blocks the ACE2 receptor mediated SARS-CoV-2 cell entry and inhibits the 3CL^pro^-mediated viral replication and infection	Khan et al. (216) Pan et al. (214)
Luteolin	Main flavonoid in honeysuckle	3CL^pro^ and cytokine storm	Preclinical	Blocks 3CLpro-mediated SARS-CoV-2 replication and infection, inhibits the cytokine storm caused by mast cells secreting proinflammatory cytokines	Theoharides (217) Shawan et al. (218)
Isorhamnetin	Flavonoid ingredient in hippophae rhamnoides	Spike protein and 3CL^pro^	Preclinical	Inhibits the 3CL^pro^ mediated SARS-CoV-2 replication and Spike protein mediated viral attachment	Zhan et al. (219) Tejera et al. (220)
Naringenin	Active ingredients of TCM	3CL^pro^, cytokine storm and ACE2	Preclinical	Inhibits the 3CL^pro^ mediated SARS-CoV-2 replication, cytokine production induced cytokine storm and ACE2 mediated viral entry	Clementi et al. (221) Maurya et al. (222) D’Amore et al. (223)
Wogonin	Active ingredients of TCM	3CL^pro^ and Akt1	Preclinical	Inhibits the 3CL^pro^ mediated SARS-CoV-2 replication and Akt1 induced infection, lung injury and lung fibrogenesis	Xia, Lu, et al. (207) Xia et al. (194)
Salvianolic acid C	Active hydrophilic compound of Danshen	Spike protein	Preclinical	Inhibits SARS-CoV-2 infection by blocking the formation of six-helix bundle core of spike protein and the binding of its RBD and ACE2	Yang et al. (224) Wang et al. (225) Hu et al. (226)
Baicalin	Active components of Scutellaria B.	3CL^pro^, RdRP and PL^pro^	Preclinical	Inhibits SARS-CoV-2 replication by interfering the 3CL^pro^, RdRP and PL^pro^	Jo et al. (227) Zandi et al. (228) Rehman et al. (229)
Baicalein	Active components of Scutellaria B.	3CL^pro^, RdRP, and Mitochondrial	Preclinical	Inhibits SARS-CoV-2 replication by interfering mitochondrial oxidative phosphorylation, ^3CLpro^ and RdRP	Huang et al. (230) Liu et al. (231) Zandi et al. (228)

### Vaccines

**Figure 5:**
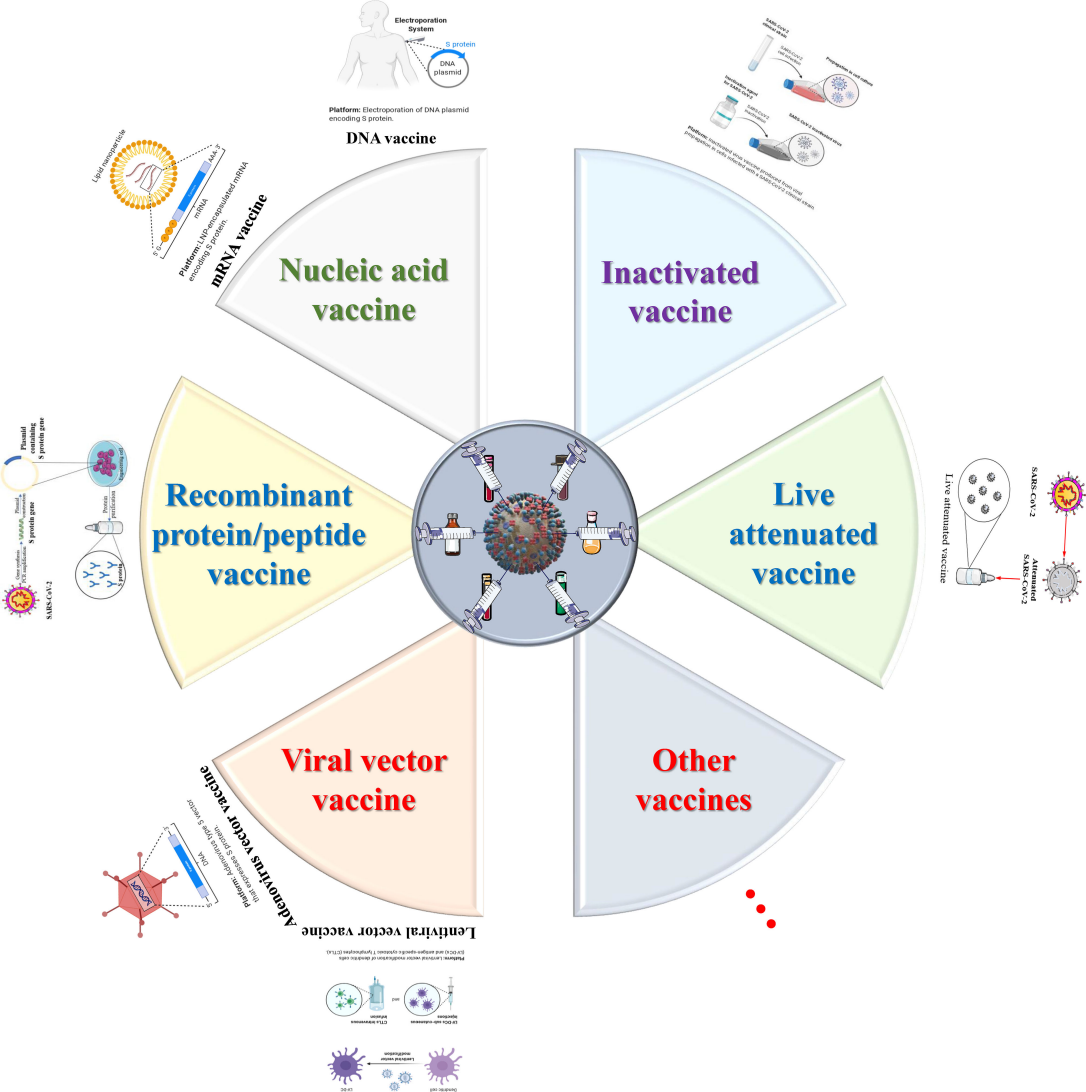
The design and development model of SARS-CoV-2/COVID-19 vaccines. One of the most important intervention strategies for COVID-19 is vaccine control. To date, six major types of vaccine candidates (live attenuated vaccines, recombinant protein/peptide vaccines, inactivated vaccines, viral vector vaccines, nucleic acid vaccines and other types of vaccines) are under development, clinical trials, authorized emergency use, and routine preventive use. These six types of candidate vaccines represent the direction of SARS-CoV-2 and even the entire coronavirus vaccine research.

The main therapeutic strategies for infectious diseases include controlling the source of infection, blocking the route of transmission and protecting the susceptible. Among them, vaccines, as an effective means to protect susceptible persons and block transmission, have always been the main weapon for humans to fight infectious diseases (234). Given that the current effective treatments against the new coronavirus are not fully recognized, the development of vaccines against SARS-CoV-2 is particularly important. At present, a variety of vaccine platforms against SARS-CoV-2 are rapidly being established and developed, including inactivated vaccines and live attenuated vaccines and viral vector vaccines and nucleic acid vaccines (DNA and mRNA) (**Figure 5**) **(** 235). With the joint efforts of scientists from all over the world, more than 322 candidate vaccines have been developed, which are in the preclinical, Phase I, Phase II through to Phase III efficacy studies and include Phase IV registered as interventional studies (https://www.who.int/publications/m/item/draft-landscape-of-covid-19-candidate-vaccines) **(** **Table 2** **)**. The rapid development of vaccine research has brought dawn to the control of the epidemic, but there are many shortcomings that need to be considered and improved.

Regarding inactivated vaccines, there are preliminary statistics of 15 such vaccines that have entered different clinical trials, including BBIBP-CorV, CoronaVac, WIBP vaccines, and Covaxin, which have entered Phase III (236). CoronaVac (Sinovac Biotech, China) can produce a wide range of neutralizing antibodies against 10 different virus strains in a variety of animals with a titer of over 90, and it has complete protection against SARS-CoV-2 infection after three immunizations (6 μg/dose) in macaques (151). Currently, CoronaVac is undergoing Phase III clinical trials in Brazil, of which 90,000 healthy participants are or will be registered. Another inactivated vaccine, covaxin, developed by an Indian Pharmaceutical company, has shown good safety and effectiveness in phase 1/2 clinical trials. At present, Covaxin is also undergoing phase III clinical trials, of which 26,000 volunteers participated (237). Other candidate inactivated vaccines are being rapidly developed in China and have been confirmed to have higher antibody titers and better safety in phase 1/2 clinical trials. Just now, a Phase III clinical trial with 15,000 participants has been launched in the United Arab Emirates (238). The development of inactivated vaccines gives us confidence in the development of vaccines against SARS-CoV-2. However, we must also recognize some of the shortcomings of inactivated vaccines and improve them. For example, the inoculation dose is large, the protection time is short, the need for multiple vaccinations, and the formation of antibody-dependent enhancement effects may aggravate viral infections.

The development of live attenuated vaccines against SARS-CoV-2 has been slow, mainly due to the limitations of the longer transformation or screening time of attenuated strains, heavy workload, and high biosafety protection standards. At present, only one attenuated influenza virus vector vaccine developed by China has entered a phase I clinical trial (ChiCTR2000037782), and three live attenuated vaccines developed by India and Turkey are undergoing preclinical evaluation (239). However, we should also realize that live attenuated vaccines can retain the complete structure of the virus and have good immunogenicity; they can simulate the natural infection process to induce humoral and cellular immunity and can produce long-lasting protection; no adjuvant is required (240). If the transformation time of attenuated strains can be optimized and biosafety is ensured, live attenuated vaccines can be an alternative direction.

At present, approximately 20 SARS-CoV-2 vaccines are being developed around the world that use the viral vector method. These vector vaccines are mainly divided into two categories: nonreplicating vector vaccines based on adenovirus and lentivirus and replicating vector vaccines based on measles, influenza, etc. The most concerning adenovirus vectors include ChAdOx1 nCoV-19/AZD1222 (Oxford University & AstraZeneca, etc., UK; D8110C00001) (241) and Ad26.COV2.S (Johnson & Johnson, USA; NCT04505722) (242), Ad5-nCoV (Academy of Military Medical Sciences & CanSino Biologics Inc, China; NCT04526990) (160) and Gam-COVID-Vac (Gamaleya, Russia; NCT04656613) (162). In addition, the 2019-nCOV candidate (Academy of Military Medical Sciences, China; ChiCTR2000031781) and defective simian adenovirus vector GRAd-COV2 (ReiThera, Italy; NCT04791423) are in phase II, with two vaccines: VXA-CoV2-1 (Vaxart, USA; NCT04563702) and hAd5-S-Fusion+N-ETSD (ImmunityBio, Inc., USA; NCT04710303) are in phase I. Lentiviral vector-based vaccines under development include LV-SMENP-DC currently in phase I/II and pathogen-specific aAPC vaccine in phase I (Shenzhen Geno-Immune Medical Institute, China; NCT04299724/NCT04276896). Phase II of the clinical trial was the intranasal influenza virus vector DelNS1-2019-nCoV-RBD-OPT1 (Beijing Wantai Biological Pharmacy, China; ChiCTR2000039715), and phase I/II of the clinical trial was the replication VSV vector rVSV-SARS-CoV-2-S/IIBR-100 vaccine (Israel Institute of Biology, Israel; NCT04608305) and three replication virus vector vaccines in Phase I: the intranasal influenza virus vector the measles vector TMV-083/V-591 (Institut Pasteur & Themis Bioscience, Austria; NCT04497298) and the VSV vector V590-001 (MSD Corp., USA; NCT04569786) and modified Ankara vector MVA-SARS-2-S (Universitätsklinikum Hamburg-Eppendorf, Germany; NCT04569383). Relying on the characteristics of few adverse reactions, good safety, and a mature production system, this type of vaccine has been developed rapidly. However, neutralizing antibodies of the vector may exist in the body, which will cause the vector to be attacked, thereby reducing the vaccine effect. Therefore, improving the effectiveness will be an important direction for the improvement of these vector vaccines.

The SARS-CoV-2 nucleic acid vaccine has quickly become the focus of vaccine research and development due to its simple development and operation, low production cost, short development and production cycle, and rapid response (243). At present, the research of such vaccines is divided into two major directions, namely, DNA vaccines and mRNA vaccines. Currently, there are 27 DNA vaccines under research in the world, 11 of which have entered the clinical trial stage, and this number will slowly increase as the technology continues to mature. ZyCoV-D is a new type of DNA vaccine candidate mainly composed of plasmid DNA loaded with the viral spike gene and signal peptide coding gene (171). The results of clinical trials (CTRI/2020/07/026352) have verified a good safety profile and induced cellular and humoral responses, which will support its further development to prevent COVID-19-related infection and death in the global population. Meanwhile, the emergency use of the ZyCoV-D vaccine in India has brought more possibilities and hopes for the development of DNA vaccines. INO-4800 is a DNA vaccine expressing S protein particles developed by Inovio Pharmaceuticals (174). Clinical trial (NCT04336410) data prove that the INO-4800 vaccine maintains one or both cells and humoral arms of the immune response for the emerging SARS-CoV-2 variant, which may be the key factor affecting the ongoing COVID-19 pandemic. Taking into account the advantages of DNA vaccines, the results of phase I clinical trials of INO-4800 (NCT04447781) and the status of entering phase II/III clinical trials (NCT04642638) once again brought great encouragement to the development of DNA vaccines. Although we have seen great hopes for DNA vaccines against the new coronavirus, we should also clearly recognize that the challenge for DNA vaccines is that they need to reach the nucleus all the way, which forces us to do more research to improve and develop a delivery system to meet the delivery efficiency of DNA vaccines (244). In addition to DNA vaccines, the development of mRNA vaccines is also in full swing. mRNA vaccines can express intracellular antigens similar to DNA vaccines, but they solve the problem of low immunogenicity of DNA vaccines and generate nonspecific immunity against the vector and delivery efficiency, so they have received more attention from researchers. Currently, two mRNA vaccines have been approved for marketing, namely BNT162b2 developed by BioNTech & Pfizer and mRNA-1273 produced by Moderna (175, 177). The results of clinical trials (NCT04368728/NCT04470427) show that the effectiveness, safety and immunogenicity of the two mRNA vaccines meet the ideal requirements. With further in-depth research on SARS-CoV-2, more mRNA vaccines have entered clinical trials, such as CVnCoV (CureVac AG; Phase II: NCT04515047), ARCT-021 (Arcturus Therapeutics, Inc.; Phase I/II: NCT04480957), LNP-nCoVsaRNA (Imperial College London; Phase I: ISRCTN17072692) and ARCoV (Academy of Military Medical Sciences; Phase I: ChiCTR2000034112), etc., and preclinical research (more than 19 candidate mRNA vaccines). Evidence from clinical trials thus far shows that mRNA vaccines are very likely to become a new platform that is fast, safe and efficient. However, to become a viable clinical alternative to traditional vaccines, mRNA vaccines must overcome two major problems related to the immunogenicity and stability of mRNA vaccines (245).

In addition to the above vaccine development strategies, recombinant protein and peptide vaccines such as human recombinant soluble ACE2 (hrsACE2), recombinant S protein nanoparticle vaccine (NVX-CoV2373), recombinant RBD protein vaccine (RBD219-N1), HR2P polypeptide and EK1C4 vaccine, etc. It can effectively induce humoral and cellular immunity to produce a wider cross-reaction, which is also an important choice for the development of SARS-CoV-2 vaccines. Each vaccine development strategy has many advantages, while at the same time, there are more or fewer shortcomings (246). The current main goal is to develop a safe and effective vaccine to curb the pandemic of SARS-CoV-2. However, we should be clearly aware that while avoiding the risks of existing vaccines, the ultimate goal of vaccine development is to develop single or mixed general vaccines for different CoVs or to establish a research and development and production platform. Only in this way can we withstand the current and future virus damage.

### Traditional Chinese Medicine

Traditional Chinese medicine (TCM) has played an important role in the prevention and treatment of infectious diseases, and its theories and methods have been traced in many classic Chinese medical works (247). Meanwhile, these TCMs achieved good results in fighting against SARS-CoV infection in 2003. Moreover, in the 74187 confirmed cases of SARS-CoV-2 infection reported in China, the effective rate of receiving TCM treatment was more than 90%, and its main effect was to significantly improve and shorten the course of disease, delay disease progression, and reduce mortality (248). At the same time, traditional Chinese medicine has also been confirmed to have a low incidence of adverse reactions and often self-healing in the treatment of COVID-19 patients (249). Given that, TCM is a valuable resource for combating the epidemic of SARS-CoV-2.

Among the abundant resources of TCM, some representative drugs have shown good anti-SARS-CoV-2 activity in terms of direct anti-virus, regulation of inflammatory immunity, and organ protection, as shown in **Figure 4**. Analysis of cytopathic effects and plaque reduction showed that the active ingredients of Lianhua Qingwen capsule significantly inhibited the replication of SARS-CoV-2 in a dose-dependent manner through Akt signaling (194). In Vero E6 cells infected with 100 TCID50 SARS-CoV-2, the IC_50_ value was 411.2 μg/mL (250). In addition, Qingfei Paidu Decoction has the effect of directly inhibiting the invasion and replication of SARS-CoV-2 by acting on the host cell ACE2 and 3CL^pro^, respectively (251). In addition, the ingredients of Huoxiang Zhengqi capsule and Xuebijing injection are reported as potential 3CL^pro^ inhibitors, which could inhibit SARS-CoV-2 replication by targeting PIK3CG and E2F1 through the PI3K/Akt pathway. Moreover, network pharmacology and molecular docking studies found that the active ingredients of multiple TCMs, including Jinhua Qinggan granules, Tanreqing injection and Huashi Baidu Decoction, can all act on replicating enzymes or host cell receptor proteins to inhibit the replication and invasion of SARS-CoV-2 (252). In addition to directly blocking the replication and invasion of SARS-CoV-2, several active ingredients in Qingfei Baidu Decoction, Xuanfei Baidu Decoction, Huashi Baidu Decoction, Jinhua Qinggan Granules, Huoxiang Zhengqi Capsules, Lianhua Qingwen Capsules, Shufeng Jiedu Capsules, Xuebi Jing injection, Reduning injection, Tanreqing injection and Shenmai injection have been proven to not only reduce inflammation and inflammatory storms but also regulate cytokines and immune dysfunction by regulating multiple signal pathway abnormalities in patients, thus alleviating SARS-CoV-2-induced COVID-19 (253). In the process of studying the damage of SARS-CoV-2 to organ function, clinical analysis found that Qingfei Paidu Decoction, Jinhua Qinggan Granules, Lianhua Qingwen Capsules, and Shufeng Jiedu Capsules may play a protective role in organ damage through the effects of expectorant, anti-inflammatory, antioxidant, and antifibrosis (248, 252).

The mechanisms of TCMs for anti-SARS-CoV-2 and organs protection are quite complicated. On this basis, it will be a great deal for the TCM treatment of SARS-CoV-2 if the specific active ingredients can be clarified. In this context, based on the TCM system pharmacology database and analysis platform (TCMSP) and literature, researchers have discovered that quercetin, kaempferol, luteolin, isorhamnetin, baicalein, naringenin, and wogonin (the latter three are in the same ranking) are the most promising important ingredients for anti-SARS-CoV-2 by comprehensive analysis using network pharmacology, bioinformation analysis, molecular docking, animal experiments, and clinical trials (252). In addition, as many as 401 compounds were found to have antiviral activity, and many ingredients have shown good therapeutic effects in experiments. A recent study found that salvianolic acid C, an active hydrophilic compound of Danshen, can effectively inhibit SARS-CoV-2 infection and block the formation of the S protein 6-HB core, with an IC_50_ value of 3.41 μmol/L (224). In a cell-based system, baicalin and baicalein, as the key active components of Scutellaria B., show strong antiviral ability by significantly inhibiting 3CL^pro^ activity, with IC_50 values_ of 10.27 and 1.69 μmol/L, respectively (254). The above findings suggest that TCM resources are very abundant, and many ingredients or compounds can be considered as lead compounds for the development of anti-SARS-CoV-2 drugs (**Table 2**). Perhaps the active ingredients of TCM can form a more promising small molecule inhibitor library in the future. Therefore, we should pay attention to and devote certain resources to screening, discovering and developing promising TCM compounds and extracts for the treatment of SARS-CoV-2.

We know that TCM prescriptions are produced in long-term exploration and practice, and their compatibility, toxicity, safety and other issues can be guaranteed. However, the abovementioned problems exist when the active ingredients and monomers of TCM are used (249, 255). We hope that TCM can be more widely used in the treatment of COVID-19, but at the same time, safety issues such as compatibility, toxicity, and adverse reactions of active ingredients and monomers of TCM should also be more studied and explored (256).

### Significant Symptomatic Therapeutic Strategy

During COVID-19, aggressive inflammation and dysfunctional immune responses are the most basic, common and important pathological features that trigger cytokine storms and mediate multiple organ system damage (257). If not well controlled, the situation will worsen and even lead to death. In severe cases, most patients experience severe lung inflammation and thrombosis (258). Therefore, anti-inflammatory and anticoagulant drugs have been proposed and implemented, including the application of low molecular weight heparin to hospitalized patients as one of the standard symptomatic therapeutic strategies (**Figure 4**). In the serum of most COVID-19 patients, the levels of proinflammatory cytokines, including IL-1β, IL-2, IL-6, IL-8, IL-17, G/GM-CSF, MCP1, CCL3 and TNF, are significantly elevated, which is considered a cytokine storm (259). Among these cytokines, IL-6 has become a stable indicator of poor prognosis and has been used in the neutralization treatment of several inflammatory diseases. Therefore, targeting serum IL-6 levels to reduce inflammation may become an important symptomatic treatment strategy (260). One clinical study (ChiCTR2000029765) showed that tocilizumab, an IL-6 receptor-targeted monoclonal antibody, could reduce the risk of severe SARS-CoV-2 infection in patients with invasive mechanical ventilation or death (261). A randomized double-blind phase III clinical trial (NCT04320615) showed that tocilizumab (8 mg/kg, intravenous injection) can significantly shorten the intensive care unit by 5.8 days (9.8 days of standard care) and shorten the discharge time by 8 days (20 days of standard care) (262). Currently, tocilizumab has registered more than 70 SARS-CoV-2-related clinical trials. CVL218 was originally discovered through a data-driven drug reuse framework that can effectively inhibit the replication of SARS-CoV-2 with an EC_50_ of 5.12 μM. In-depth studies have shown that CVL218 (1 and 3 μM) treatment for 12 h can significantly reduce the production of IL-6 by 50% and 73% in peripheral blood mononuclear cells induced by CpG (microbial DNA sequence containing unmethylated CpG dinucleotides), respectively. *In vivo* studies have shown that CVL218 is mainly distributed in lung tissues and has no obvious toxicity (263). The above results suggest that CVL218 has a significant anti-inflammatory cytokine effect on SARS-CoV-2-induced immunopathological symptoms. Based on this, we think that targeted intervention of inflammatory cytokines is an important SARS-CoV-2 treatment strategy that can be studied in depth.

Multiple studies suggest that excessive inflammatory production of proinflammatory cytokines such as IL-6 and TNF-α may trigger ARDS, which will accelerate disease progression and increase the risk of death in COVID-19 patients (264, 265). Therefore, controlling the development of ARDS may also be a feasible treatment strategy for COVID-19. At present, a number of clinical studies (NCT04244591/NCT04327401/NCT04476992/NCT04306393…) are using strategies such as glucocorticoids, small molecule drugs, recombinant interferon and NO inhalation to explore the effectiveness of intervening in ARDS to affect COVID-19 (266, 267). Perhaps this strategy will provide more evidence for the safety and efficacy of treating COVID-19.

Immunomodulators are an important class of substances that affect the function of the immune system. Among them, pegylated interferon-α, which is approved for the treatment of hepatitis B/C viruses (HBV/HCV), can be used to stimulate the innate antiviral response of patients infected with SARS-CoV-2 (ChiCTR2000029387) (268). A retrospective study showed that pegylated interferon-α aerosol (5 million IU, bid) and arbidol (600 mg/day) treatment can significantly reduce the upper respiratory tract viral load and shorten the time for the inflammatory response indicators (IL-6 and CRP) in blood to return to normal with no obvious adverse reaction (269). Meanwhile, some clinical trials evaluated the therapeutic effect of glucocorticoids and found that they can significantly reduce the cytokine storm and relieve the corresponding tissue damage, which is beneficial to the treatment of severe COVID-19 (reduced 1/3 of mortality rate in patients using ventilators) and may affect the clearance of the virus in mild patients (270). The above results indicate that the use of immunomodulators to affect immune function will be a symptomatic treatment strategy for COVID-19 that can be considered. Although it is not given priority, the indications, dosage and course of treatment can be strictly controlled in consideration of the patient’s situation to ensure the maximum benefit of the patient.

The use of antibodies contained in the plasma of convalescent patients to suppress viremia for passive immunotherapy is considered to be a promising option for anti-SARS-CoV-2 infection. Currently, there have been clinical trials to test the effectiveness of plasma in recovering patients, and a study showed that the mortality rate of patients receiving convalescent plasma therapy is significantly lower than that of patients not receiving plasma therapy (271). *In vitro* experiments showed that antibodies in the serum of SARS-CoV-2-infected patients can effectively neutralize SARS-CoV-2. Moreover, clinical trials of administering convalescent plasma to 5,000 COVID-19 hospitalized patients in the early stages have also proven to be safe because the incidence of serious adverse events is very low (272). Therefore, convalescent plasma seems to be a good symptomatic treatment strategy in the case of solving the problems of ionomer safety and whether it needs a different storage method from ordinary plasma.

In addition, blood purification, NK-cell therapy, MSC transplantation therapy and Treg cell therapy have also been mentioned and are being studied. These therapies mainly alleviate and eliminate the pathological symptoms of patients, including inflammation, immune dysfunction, organ failure, etc., by adjusting immune function, removing inflammatory cytokines from the body, and directly killing SARS-CoV-2 infections (186). Changes such as lymphopenia and increased inflammatory cytokines in COVID-19 patients can induce symptoms of inflammation, immune function, and organ system dysfunction, which can be considered potential biomarkers and intervention targets for disease progression (273). Therefore, symptomatic treatments such as improving lymphopenia, reducing inflammation, and regulating immunity will become promising treatment strategies.

## Perspectives and Conclusions

The continuous outbreak of SARS-CoV-2 and the endless emergence of new mutant strains once again emphasize the urgency of continuing to explore, screen, and prevent COVID-19 globally. All this urgently requires precise target determination and mechanism elucidation in order to develop specific or broad-spectrum drugs for SARS-CoV-2 virus entry, replication, pathological changes or prevention.

While exploring and determining the effective targets for fighting against SARS-CoV-2, we should highly combine the experience of the three CoV pandemics, clarify the SARS-CoV-2 genome and structural information, and lay the foundation for screening targets; comprehensively consider the pathophysiological characteristics and mechanisms of viral entry, replication, assembly, infection and pathogenic processes to accurately analyze the crystal structure of related enzymes and proteins, and provide direct evidence for the target; and combine omics, bioinformatics, computer virtual screening and artificial intelligence and other technologies to explore, screen and confirm targets with maximum efficiency.

Furthermore, the development of vaccines and drugs needs to be carried out at multiple levels. Specifically, considering the lethality and disability of COVID-19, short-term research focuses on “old drugs and new use”, rapid screening of FDA-approved drugs and clinical trials, and cooperation with other medication considerations to speed up the treatment of patients. After multichannel experience accumulation, developing innovative drugs targeted at different populations with good activity and selectivity against viruses through virtual screening and computer drug design, candidate drug preclinical research, and corresponding protective measures are key to future prevention and treatment. Moreover, it is necessary to minimize the occurrence and impact of drug resistance to maintain the efficacy of these innovative drugs; from a long-term perspective, broad-spectrum anti-CoV drugs should be developed to provide sufficient R&D experience and test platforms for possible future outbreaks.

Currently, there are only a few clinically approved drugs, vaccines and corresponding therapeutic strategies for COVID-19, and we cannot control the long-term consequences. Therefore, through the existing vaccination prevention, contact tracing, isolation of infected persons, and effective supportive treatment of SARS-CoV-2-infected persons, the diagnosis of symptomatic and asymptomatic persons and their close contacts as soon as possible is still the key means to prevent the further spread and control the disease. Furthermore, we should also realize that focusing on international cooperation and sharing anti-epidemic experiences will provide new impetus for the dissemination and confirmation of treatment strategies.

## Author Contributions

HZ and W-JN contributed to the conception or design of the review. HZ and W-JN wrote the manuscript. WH, MC, ZW, and Y-CS collected and analyzed the latest literature and intelligence on the pandemic. W-JN, MC, and ZW revised the original draft. W-JN, MC, and Y-CS critically reviewed and edited the latest version for important intellectual content. All authors contributed to the article and approved the submitted version.

## Funding

This work was supported by the Science Foundation of Anhui Provincial Cancer Hospital (No. 2020YJQN008), the National Natural Science Foundation of China (No. 81803602), the Natural Science Foundation of Anhui Province (No. 1708085QH207), and the Fundamental Research Funds for the Central Universities (No. WK9110000018).

## Conflict of Interest

The authors declare that the research was conducted in the absence of any commercial or financial relationships that could be construed as a potential conflict of interest.

## Publisher’s Note

All claims expressed in this article are solely those of the authors and do not necessarily represent those of their affiliated organizations, or those of the publisher, the editors and the reviewers. Any product that may be evaluated in this article, or claim that may be made by its manufacturer, is not guaranteed or endorsed by the publisher.
